# Supplementary figures and images for: Structural insights into loss of function of a pore forming toxin and its role in pneumococcal adaptation to an intracellular lifestyle
Source: PLoS Pathog. 2020 Nov 20;16(11):e1009016. doi: 10.1371/journal.ppat.1009016 (PMC7717573; doi:10.1371/journal.ppat.1009016)

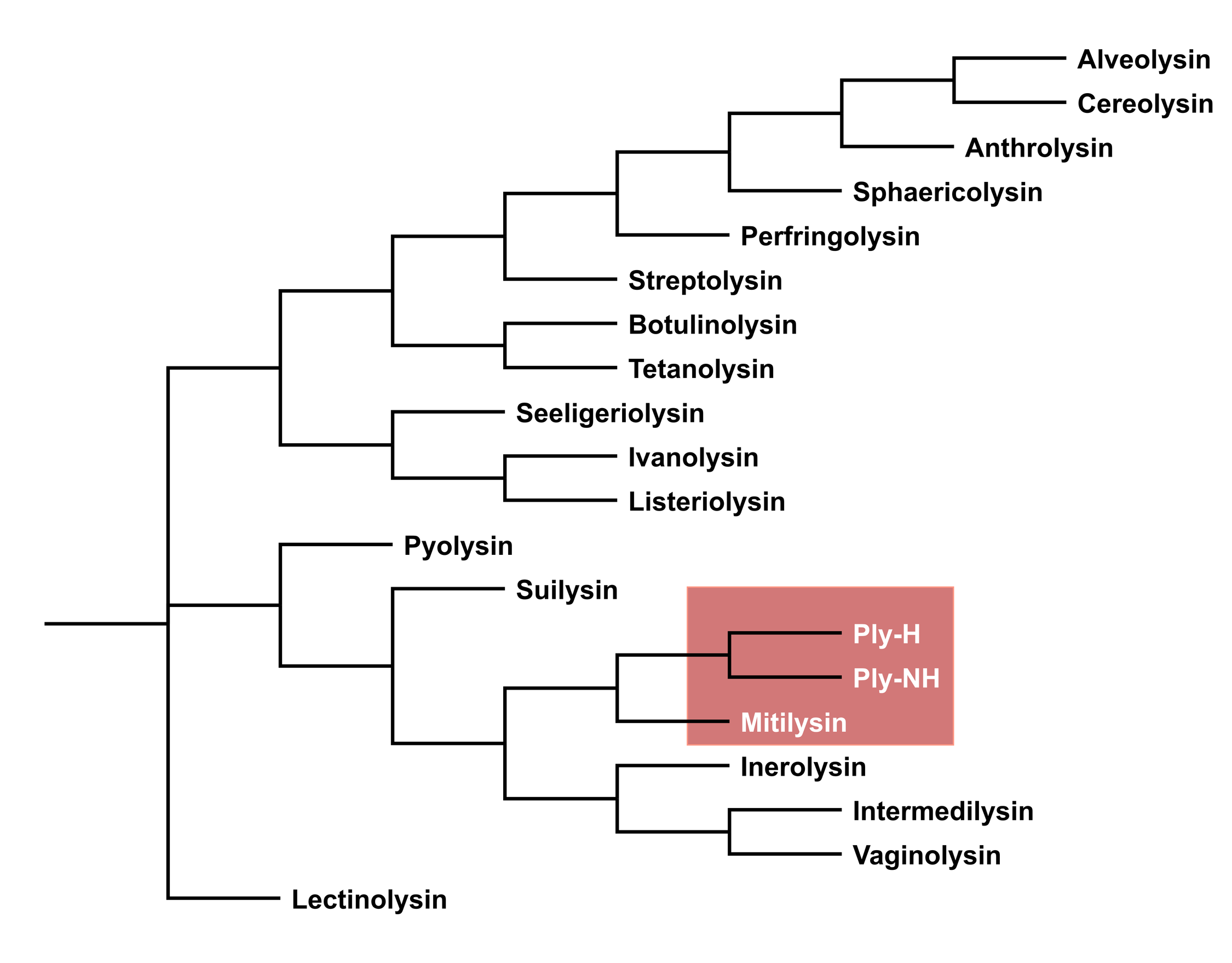

Supplement: S1 Fig — The evolutionary history was inferred using the Maximum Parsimony method. The bootstrap consensus tree inferred from 500 replicates is taken to represent the evolutionary history of the taxa analyzed. Evolutionary analyses were conducted in MEGA7. Gene Bank Accession numbers: Ply-H:AAK75991.1, Ply-NH:ABO21379.1, Mitilysin:ABK58695.1, Suilysin:CAC94851.1, Intermedilysin:BAA89790.1, Vaginolysin:ACD39461.1, Inerolysin:WP_009310637.1, Ivanolysin:AQY45513.1, Listeriolysin:CAA42639.1, Seeligeriolysin:CAA42996.1, Pyolysin:AAC45754.1, Botulinolysin:BAV54146.1, Tetanolysin:SUY56616.1, Streptolysin:NP_268546.1, Perfringolysin:WP_126964861.1, Alveolysin:EEL68223.1, Sphaericolysin:BAF62176.1, Cereolysin: AAX88798.1, Anthrolysin:RVU61618.1, Lectinolysin: EHE47793.1 (TIF) [file ppat.1009016.s001.tif]

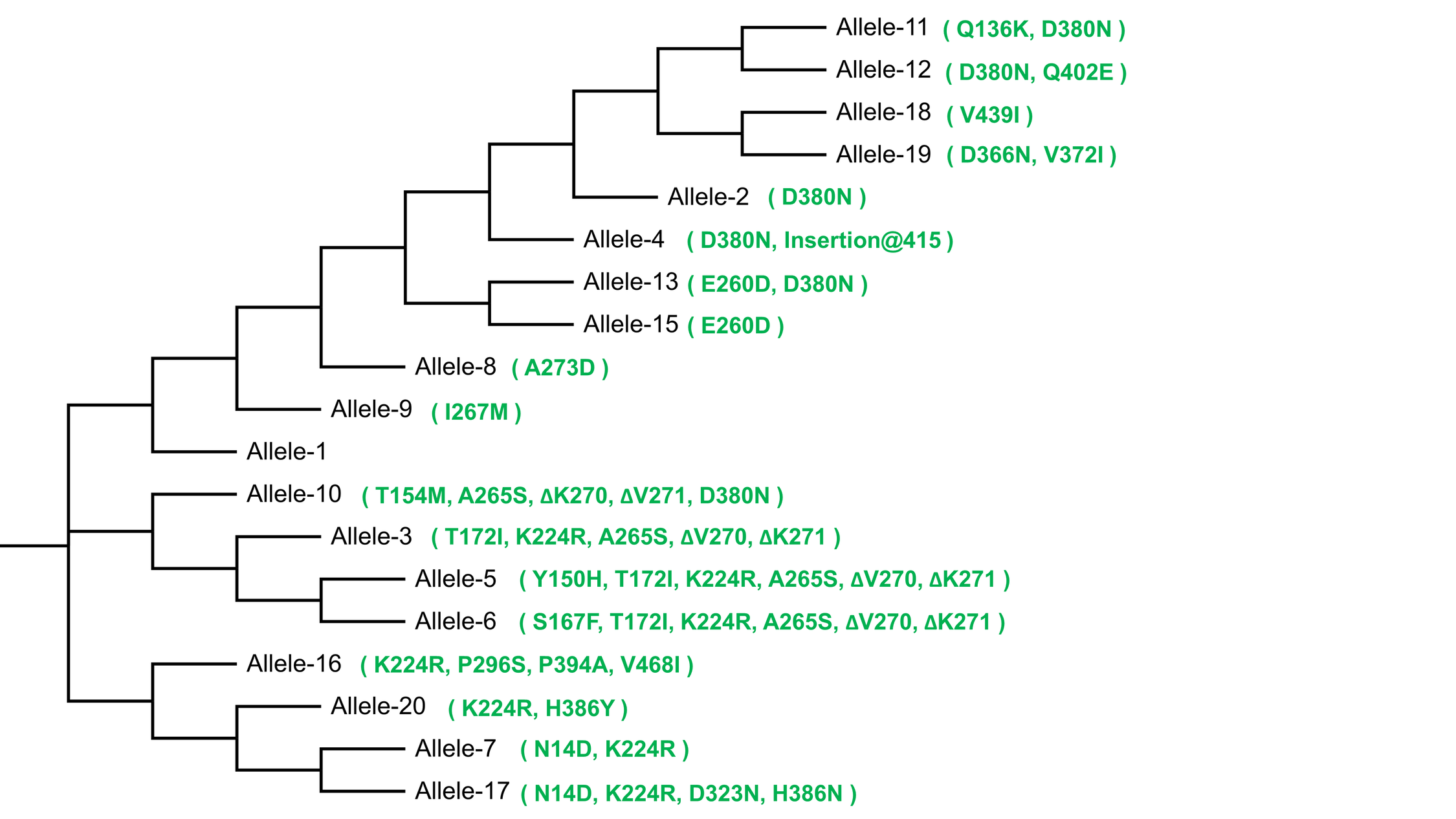

Supplement: S2 Fig — The evolutionary history was inferred using the Maximum Parsimony method. The bootstrap consensus tree inferred from 500 replicates is taken to represent the evolutionary history of the taxa analyzed. Evolutionary analyses were conducted in MEGA7. The mutations with respect to the wild type allele-1 are mentioned in parenthesis. Gene Bank Accession numbers: Allele-1:GU968409.1, Allele-2:GU968411.1, Allele-3:EF413957.1, Allele-4:EF413925.1, Allele-5:EF413960.1, Allele-6:EF413939.1, Allele-7:EF413936.1, Allele-8:GU968401.1, Allele-9:GU968397.1, Allele-10:EF413956.1, Allele-11:EF413933.1, Allele-12:EF413929.1, Allele-13:EF413924.1, Allele-15:GU968405.1, Allele-16:GU968252.1, Allele-17: GU968340.1, Allele-18: GU968232.1, Allele-19: KP982898.1, Allele-20 [17]. (TIF) [file ppat.1009016.s002.tif]

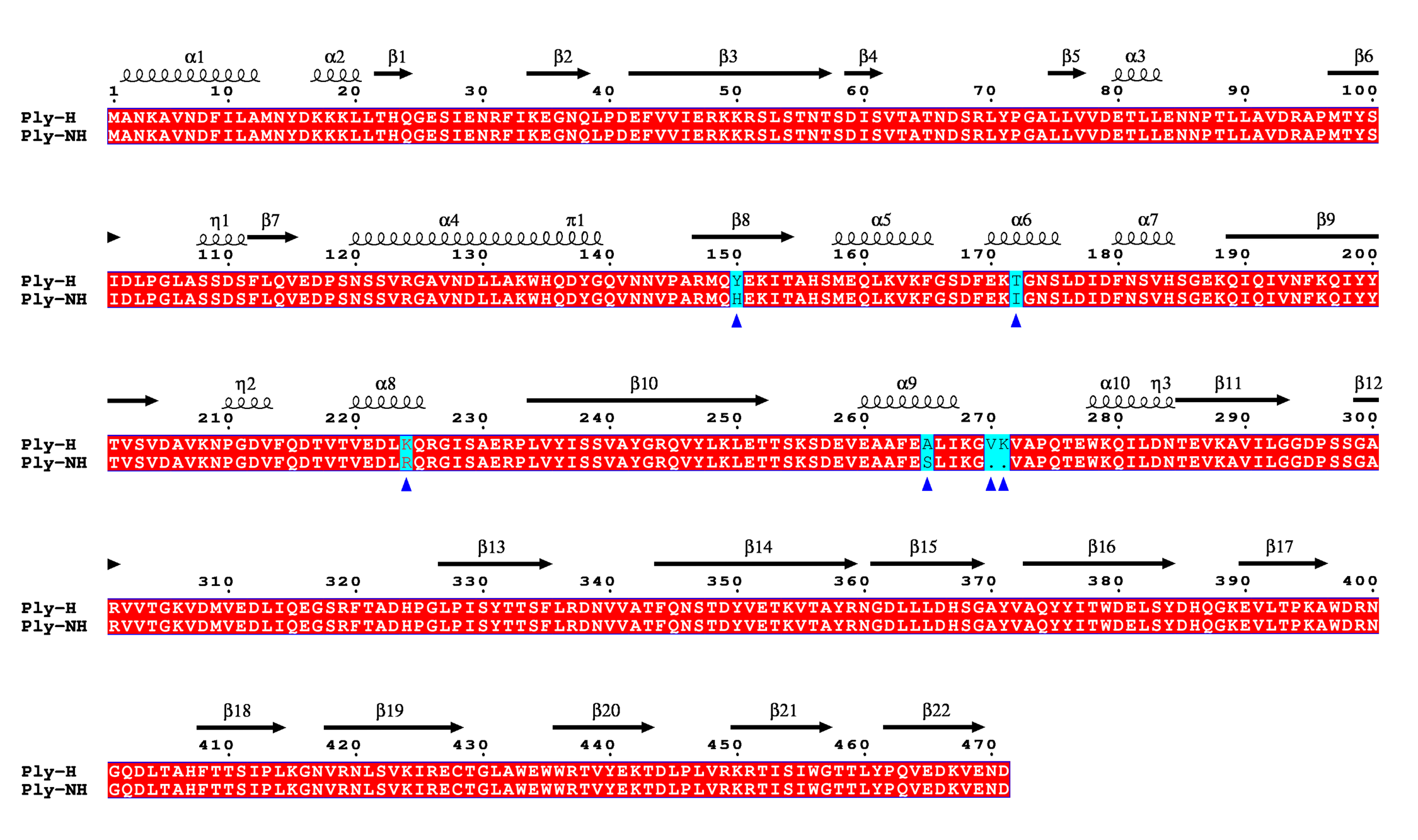

Supplement: S3 Fig — The amino acid sequence of Ply variants have been aligned using Clustal W. Invariant residues are highlighted in red boxes while deletion and substitutions are showed in cyan color with blue triangles. The secondary structural elements are shown for the crystal structure of Ply-NH. The figure was prepared in ESpript. (TIF) [file ppat.1009016.s003.tif]

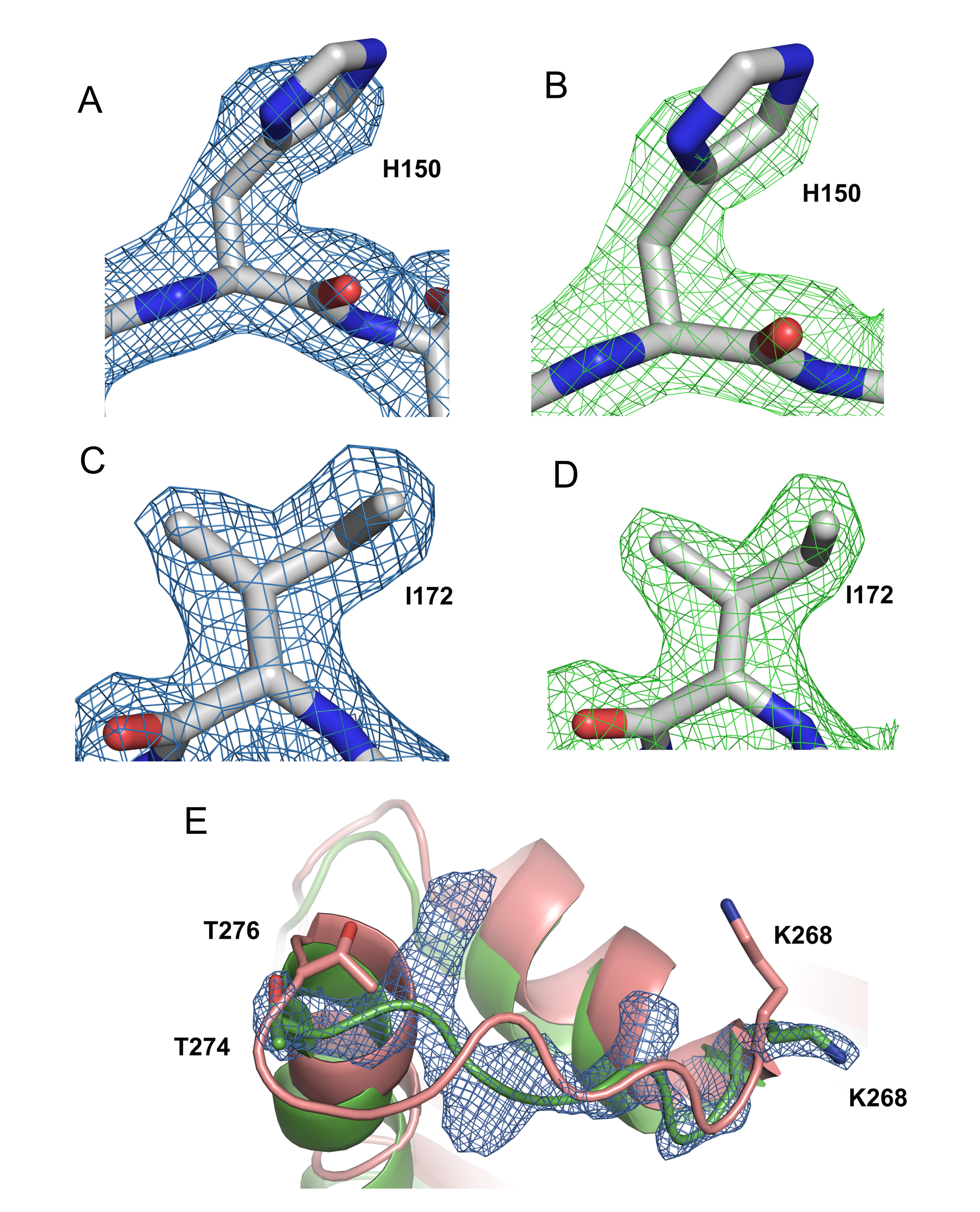

Supplement: S4 Fig — Two key substitutions and one deletion region in Ply-NH. (A) 2Fo-Fc map (blue color) for H150. (B) Fo-Fc omit map (green color) for H150. (C) 2Fo-Fc map (blue color) for I172. (D) Fo-Fc omit map (green color) for I172. (E) The 2Fo-Fc map (blue color) for the loop which has the deletion. (TIF) [file ppat.1009016.s004.tif]

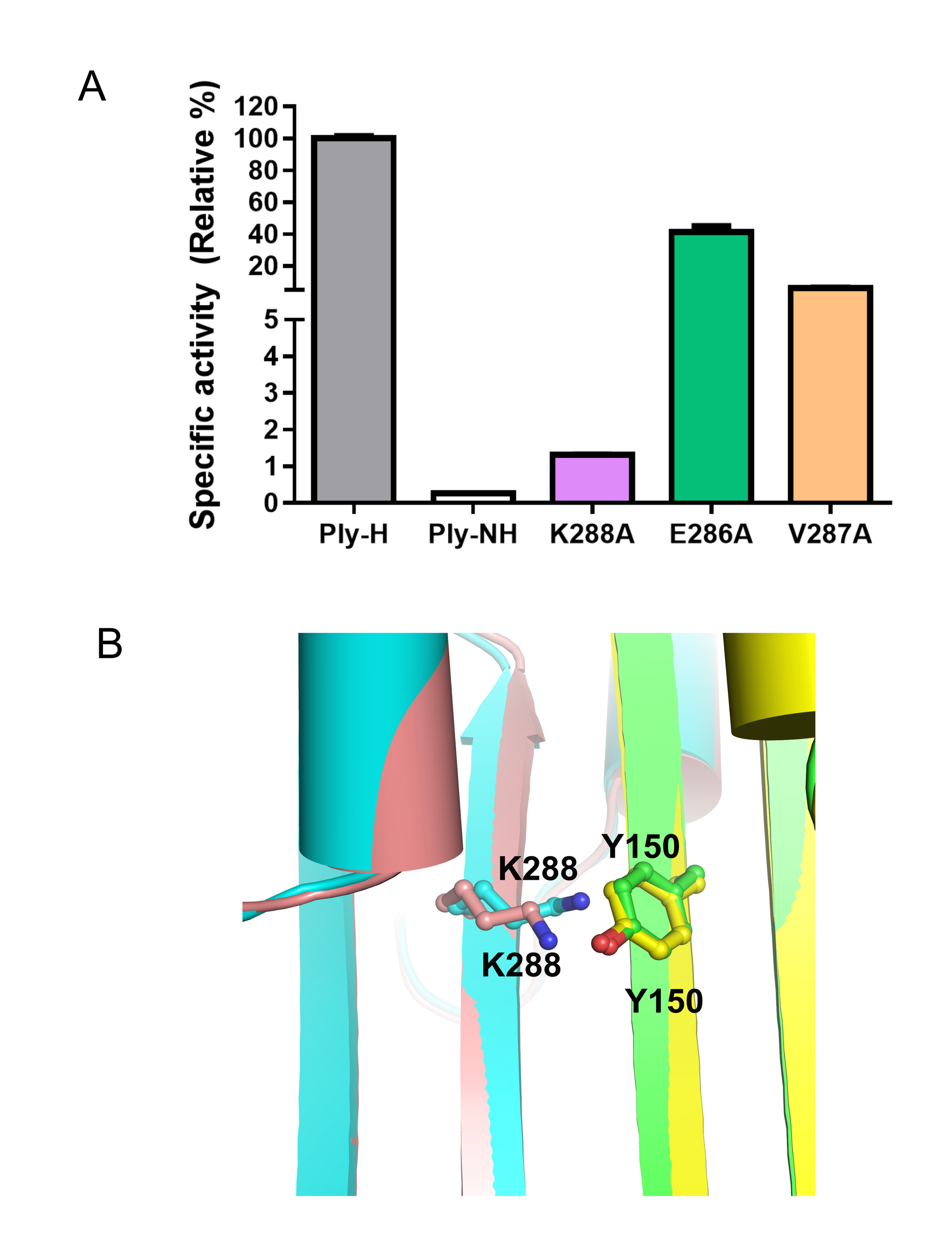

Supplement: S5 Fig — (A) Specific hemolytic activity of Ply-H mutants indicating the importance of K288 residue in the pore formation through cation-π interaction, expressed as percentage relative to Ply-H. Data is presented as mean ± SD of triplicate wells. (B) Structural superposition of pore-form of Ply-H and mitilysin (from Streptococcus mitis) demonstrating conservation of cation-π interaction. Mitilysin pore-form model was generated using Ply (5LY6) as template. The Ply-H monomers are shown in cyan and green color, while mitilysin monomers are in brown and yellow. The residue side chains are represented in ball and stick and protein molecule as cartoon. (TIF) [file ppat.1009016.s005.tif]

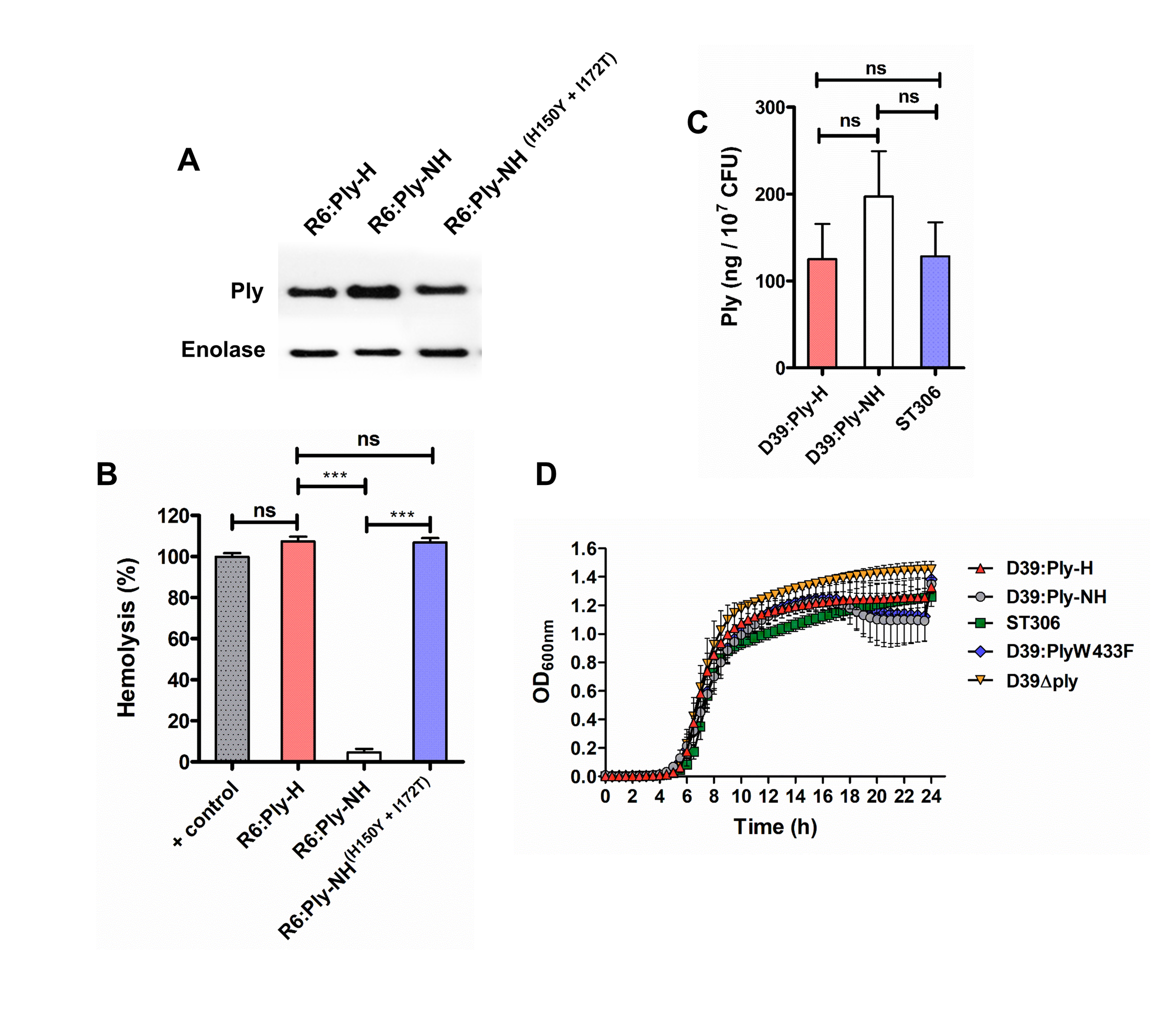

Supplement: S6 Fig — (A) Western blot using anti-Ply and anti-Enolase (house-keeping gene) antibody to demonstrate similar expression levels of Ply across different SPN R6 strains. (B) Hemolysis assay of SPN R6 lysates expressed as percentage activity relative to positive control (0.05% triton X-100). (C) ELISA-determined Ply production per 107 bacteria in D39:Ply-H, D39:Ply-NH and ST306. (D) Growth curves of different SPN strains measured by capturing optical density at 600 nm at different time points. Data information: Data is presented as mean ± SD of triplicate wells (B-C) or samples (D). Statistical analysis was performed using one-way ANOVA with Tukey’s multiple comparison test (B-C). ns, non-significant, ***p<0.001. (TIF) [file ppat.1009016.s006.tif]

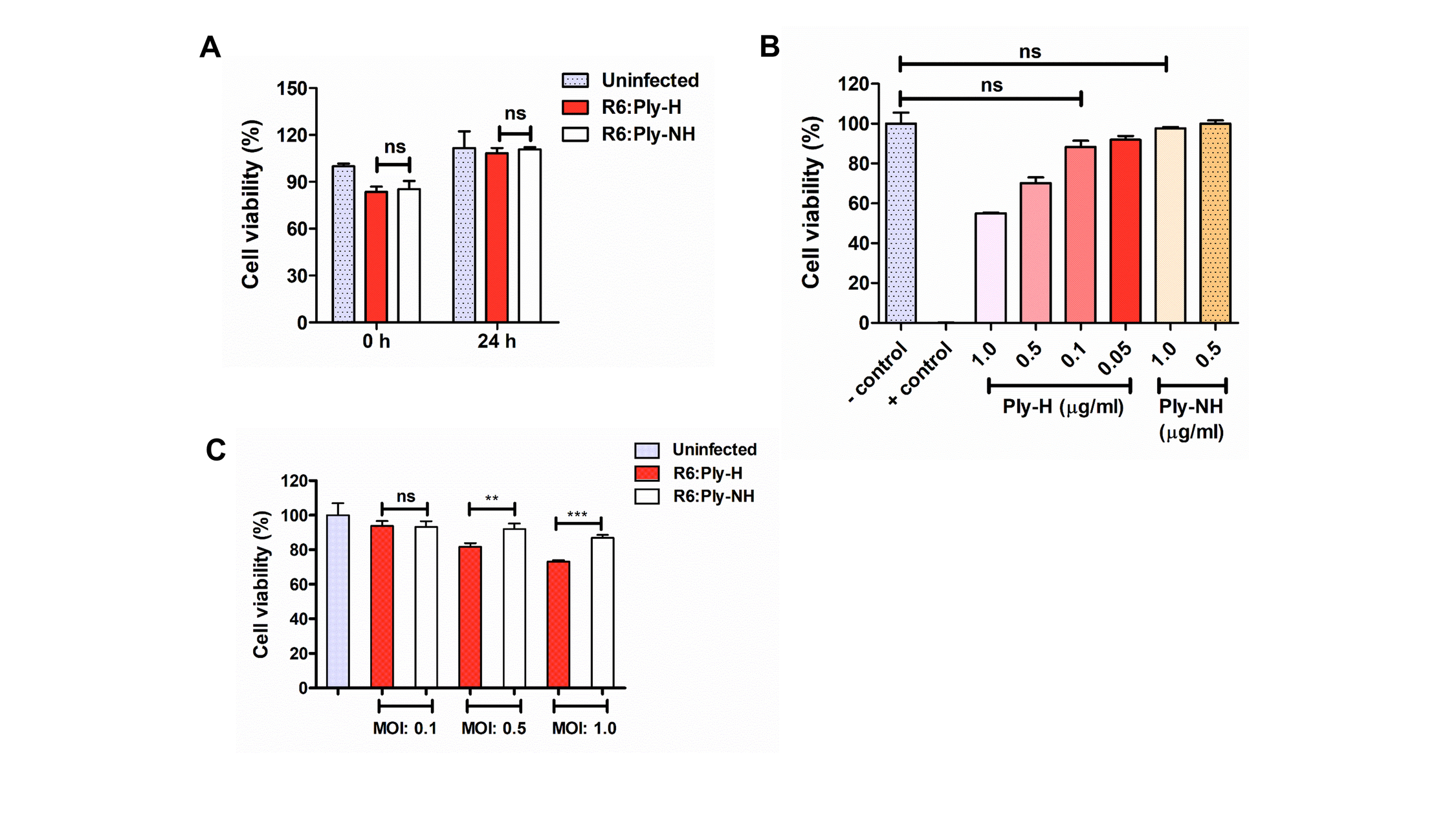

Supplement: S7 Fig — (A) A549 cell viability assay performed at 0 h and 24 h following infection with R6:Ply-H and R6:Ply-NH. (B) A549 cell viability assay performed using different concentrations of Ply-H (0.05 to 1 μg/ml) and Ply-NH. (C) THP-1 cell viability assay performed at 9 h following infection with indicated MOIs of R6:Ply-H and R6:Ply-NH. Data information: Uninfected cells and cells treated with 0.05% triton X-100 were taken as negative and positive controls, respectively. Experiments are performed thrice and data of representative experiments are presented as mean ± SD of triplicate wells. Statistical analysis was performed using Student’s two-tailed unpaired t-test (A, C) or one-way ANOVA with Tukey’s multiple comparison test (B). ns, non-significant; *p<0.05; **p<0.01; ***p<0.001. (TIF) [file ppat.1009016.s007.tif]

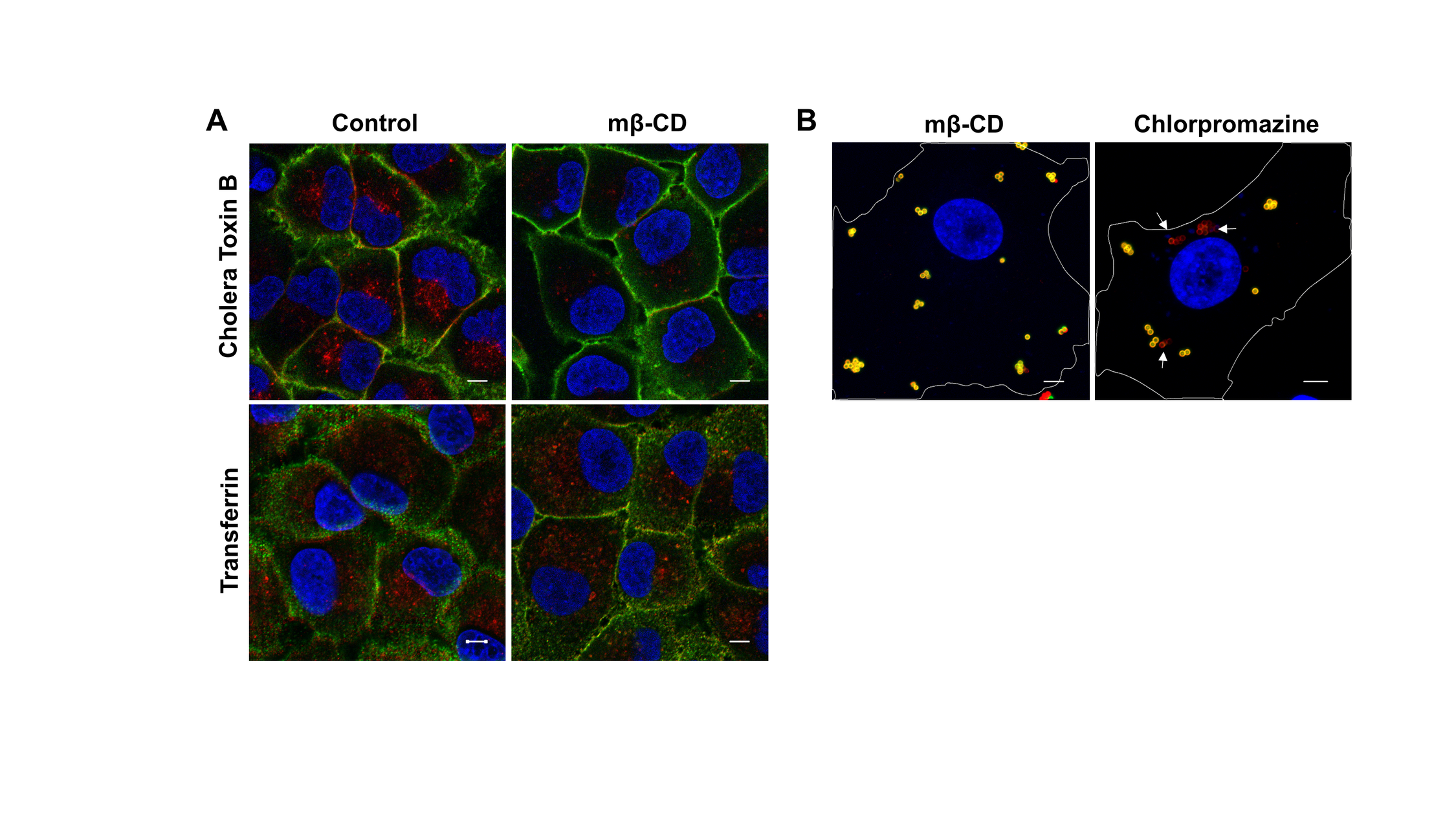

Supplement: S8 Fig — (A) Inhibition of cholera toxin B (CtxB), a lipid raft pathway specific cargo, uptake by A549 cells following treatment with Mβ-CD (5 mM, 1 h). Transferrin, a clathrin dependent endocytosis pathway specific cargo was used as negative control. Scale bar: 5 μm. (B) Internalization of CtxB coated latex beads by A549 cells following pre-treatment with the clathrin endocytosis inhibitor CPZ (15 μM, 1 h) and lipid raft endocytic pathway inhibitor Mβ-CD (5 mM, 1 h). Internalized beads are shown in red (arrow mark) while external beads are dual (yellow) colored. Scale bar: 5 μm. (TIF) [file ppat.1009016.s008.tif]

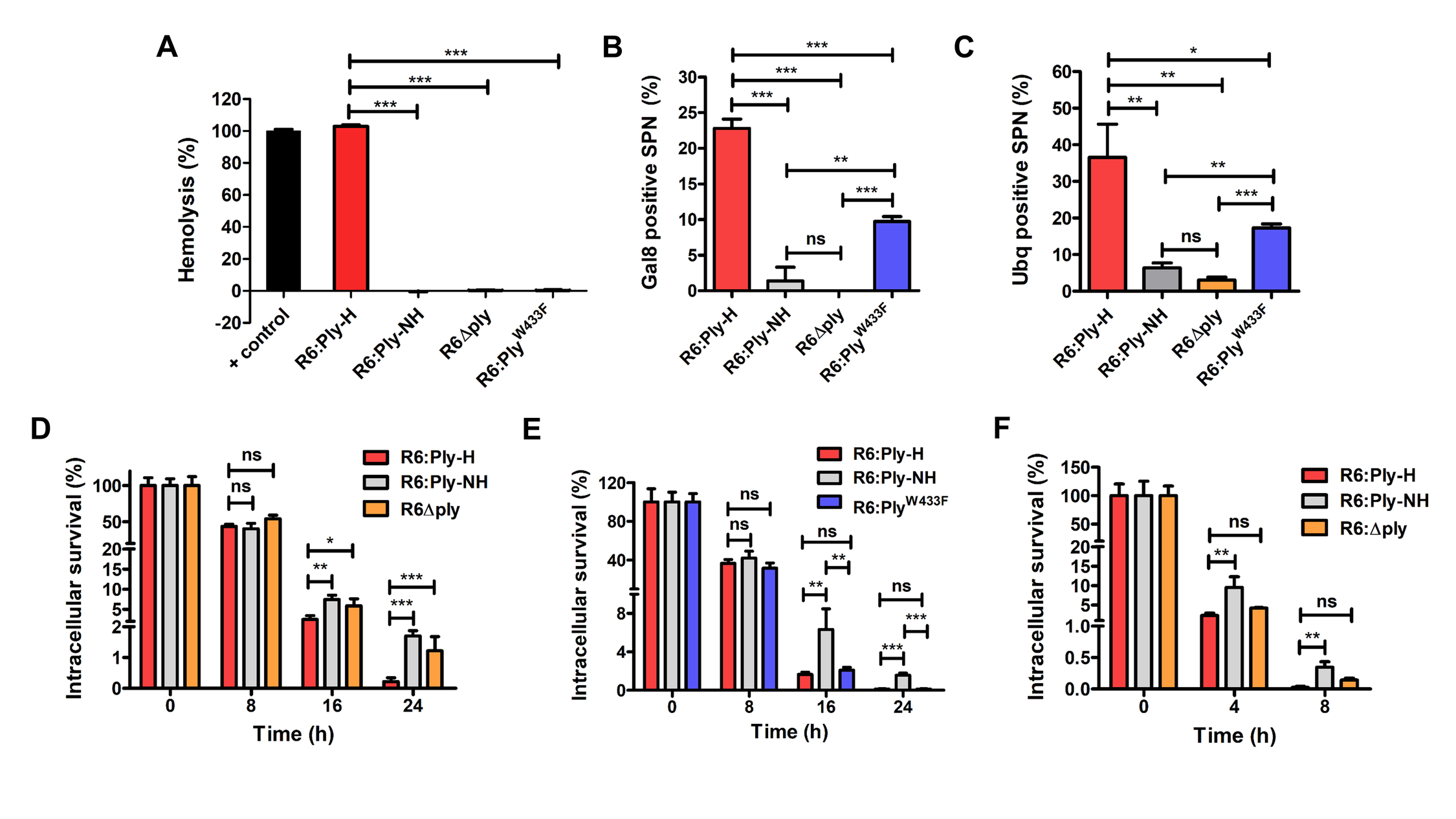

Supplement: S9 Fig — (A) Hemolysis assay of SPN R6 lysates expressed as percentage activity relative to positive control (0.05% triton X-100). (B) Percentage co-localization of Gal8 with SPN R6 strains expressing either Ply-H, Ply-NH, Δply or PlyW433F in A549s at 18 h.p.i. (C) Percentage co-localization of Ubq with SPN strains expressing either Ply-H, Ply-NH, Δply and PlyW433F in A549s at 18 h.p.i. (D, E) Intracellular survival efficiency of SPN strains expressing either Ply-H, Ply-NH and Δply mutant (D) or Ply-H, Ply-NH, PlyW433F (E) in A549s were calculated as percent survival at indicated time points relative to 0 h. (F) Intracellular survival efficiency of SPN strains expressing either Ply-H, Ply-NH or Δply mutant in THP-1s were calculated as percent survival at indicated time points relative to 0 h. Data information: Experiments are performed thrice and data of representative experiments are presented as mean ± SD of triplicate wells. Statistical analysis was performed using one-way ANOVA with Tukey’s multiple comparison test (A-F). ns, non-significant; *p<0.05; **p<0.01; ***p<0.001. (TIF) [file ppat.1009016.s009.tif]

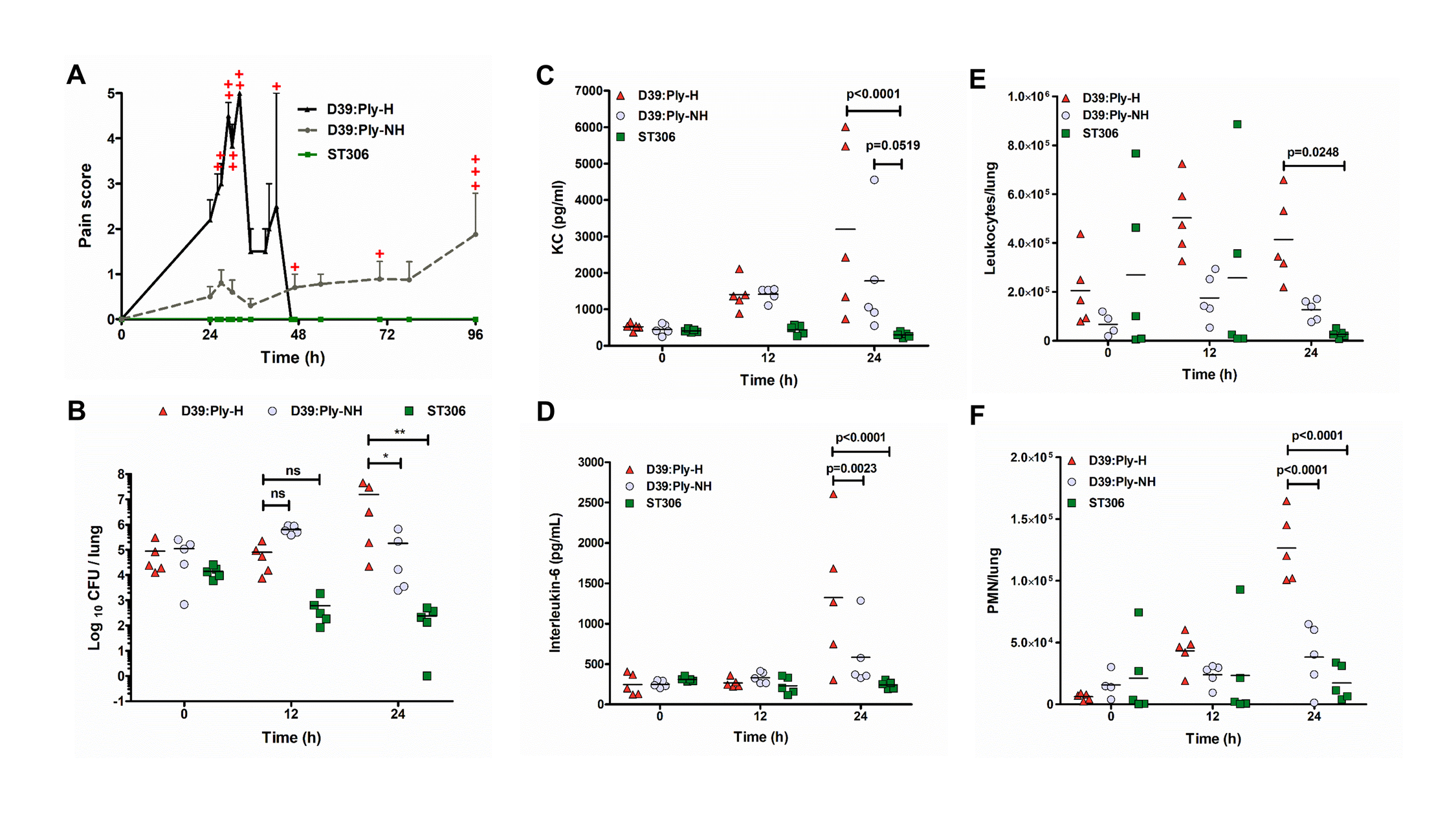

Supplement: S10 Fig — (A) Pain score, according to the scheme of Morton, in CD1 mice infected with 1.5×10^6^ CFU SPN in 50 μl PBS. Red crosses indicate where a mouse was culled due to ill health. n = 10 mice per group. (B) Lung CFU over the first 2 days of infection. ELISA-determined KC (C) and interleukin-6 (D) concentrations in lung homogenates from infected mice. (E) Numbers of CD45+ leukocytes and (F) CD45+, Gr-1+, F4/80 low neutrophils (PMN) in lung homogenates from infected mice as determined by flow cytometry. p-values in (C-F) are from two-way ANOVA analysis with Dunnett’s multiple comparisons test; n = 5 mice per group (B-F). (TIF) [file ppat.1009016.s010.tif]

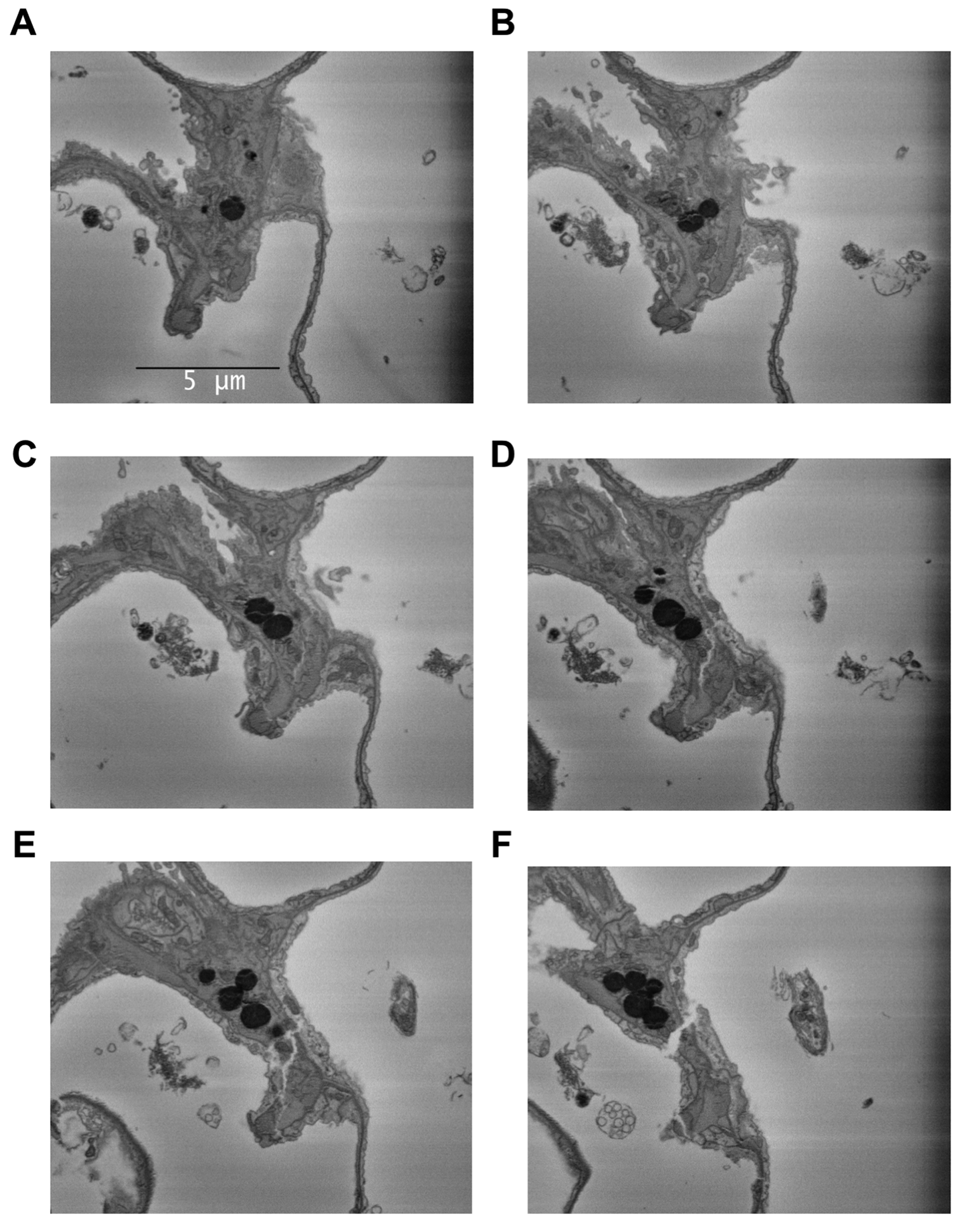

Supplement: S11 Fig — (A-F) Electron microscopy images of ST306-infected lungs. A single mouse was infected with 1.5×10^6^ CFU ST306 in 50 μl PBS through intranasal route and culled at 24 h.p.i. Fixative-perfused lungs were removed and embedded in resin before imaging on a serial block face scanning electron microscope (SBF-SEM). Images in (A-F) are sequential planes 180 nm apart, covering a total depth of 1080 nm. Scale bar: 5 μm. (TIF) [file ppat.1009016.s011.tif]

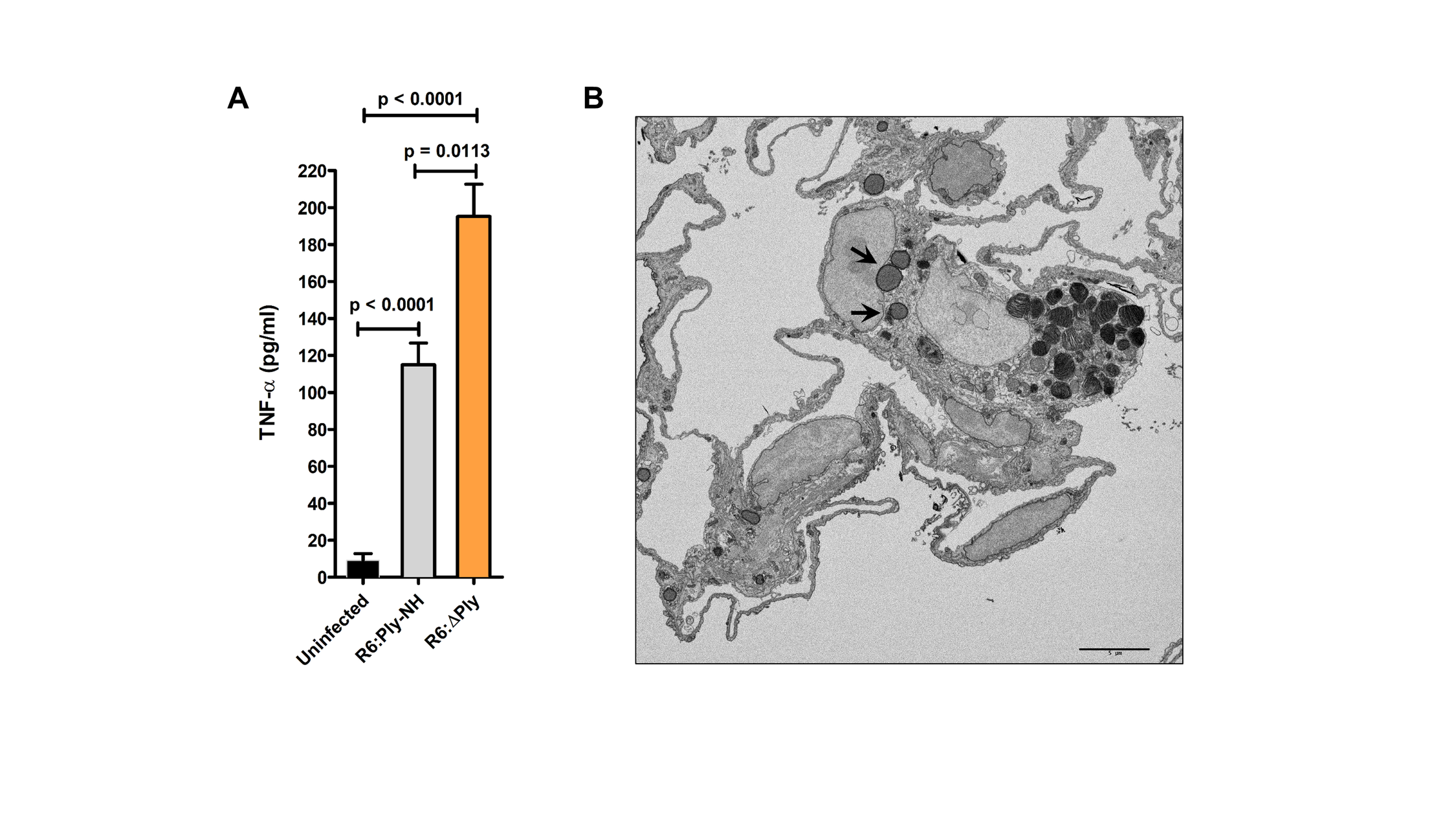

Supplement: S12 Fig — (A) Healthy donor dendritic cells were infected with pneumococci, at MOI 1, for 24 h and TNF-α was detected in culture supernatant by ELISA. Data are from n = 3 donors with two technical replicates per donor. p-values are from one-way ANOVA with Tukey’s multiple comparison test. (B) SBF microscopy image of D39:Ply-NH-infected lung. Mouse infected with 1.5×10^6^ CFU D39:Ply-NH in 50 μl PBS through intranasal route was culled at 24 h.p.i. Fixative-perfused lungs were removed and embedded in resin before imaging on a SBF-SEM. A representative image of all the images used in S1 Movie is shown. Scale bar: 5 μm. (TIF) [file ppat.1009016.s012.tif]

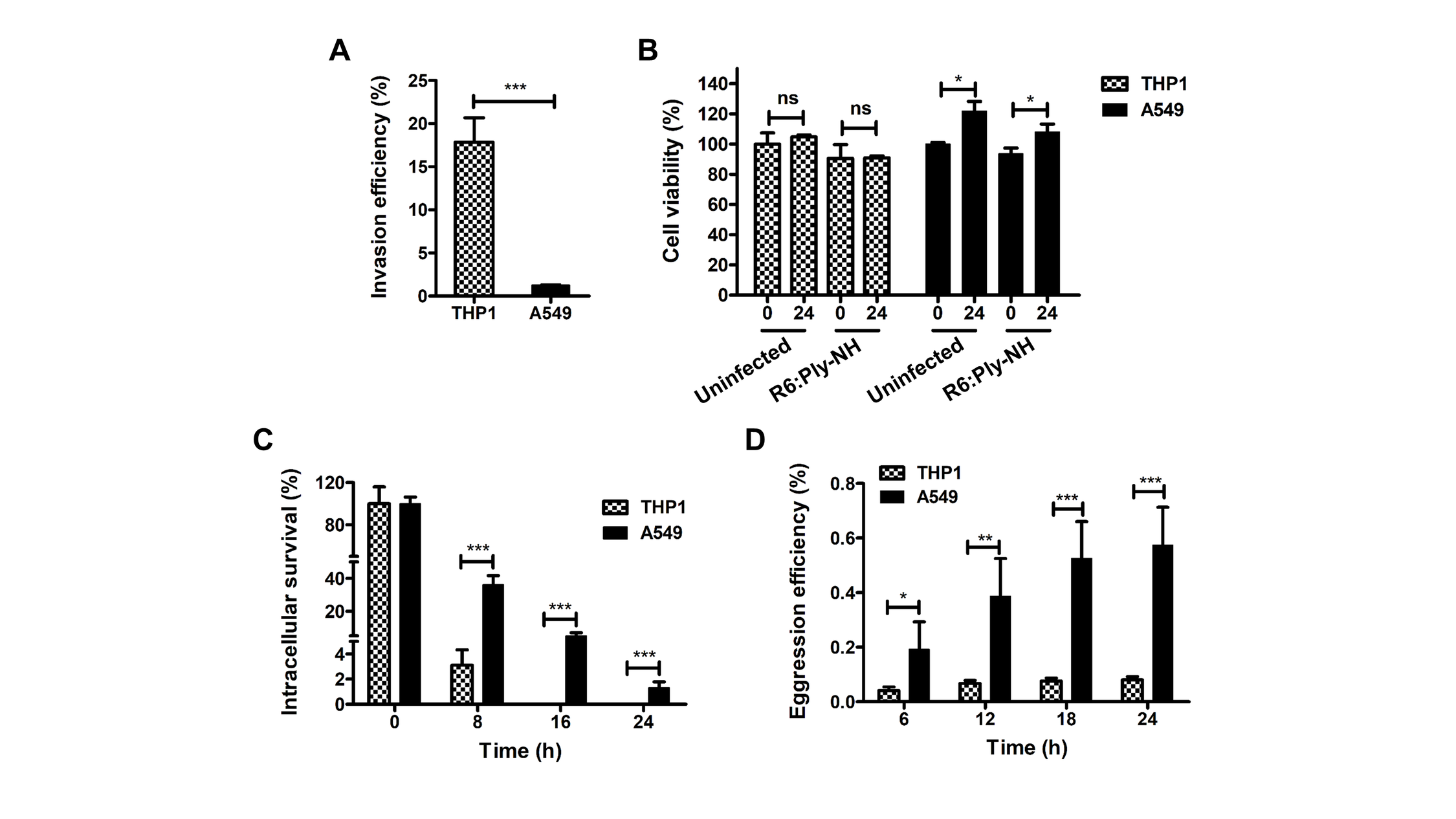

Supplement: S13 Fig — (A) Comparison of invasion efficiency of R6:Ply-NH in THP-1 macrophages and A549 cells. (B) Comparison of viability of THP-1 and A549 cells performed at 0 h and 24 h following infection with R6:Ply-NH. Uninfected cells were taken as negative control (C) Comparison of intracellular survival efficiency of R6:Ply-NH in THP-1 and A549 cells, calculated as percent survival at indicated time points relative to 0 h. (D) Comparison of egression efficiency of R6:Ply-NH in THP-1 and A549 cells, calculated as percent egression at indicated time points relative to 0 h. Data information: Experiments are performed twice and data of representative experiments are presented as mean ± SD of triplicate wells. Statistical analysis was performed using Student’s two-tailed unpaired t-test (A-D). ns, non-significant; *p<0.05; **p<0.01; ***p<0.001. (TIF) [file ppat.1009016.s013.tif]
