# Supplementary material for: Structural insights into loss of function of a pore forming toxin and its role in pneumococcal adaptation to an intracellular lifestyle
Source: PLoS Pathog. 2020 Nov 20;16(11):e1009016. doi: 10.1371/journal.ppat.1009016 (PMC7717573; doi:10.1371/journal.ppat.1009016)
Supplement: S3 Table — (DOCX) [file ppat.1009016.s016.docx]

**S3 Table. List of *S. pneumoniae* strains.**

| **STRAIN** | **SOURCE** |
| --- | --- |
| D39 (Serotype 2, encapsulated) | Mitchell TJ, Univ. of Birmingham, UK |
| R6 (Serotype 2, non-encapsulated) | Mitchell TJ, Univ. of Birmingham, UK |
| TIGR4 (Serotype 4, encapsulated) | Mitchell TJ, Univ. of Birmingham, UK |
| 01-1956 (Serotype 1, ST306, encapsulated) | Mitchell TJ, Univ. of Birmingham, UK |
| D39:Ply-H | This study |
| D39:Ply-NH | This study |
| D39Δ*ply* | This study |
| D39:Ply^W433F^ | This study |
| R6:Ply-H | This study |
| R6:Ply-NH | This study |
| R6:Ply-DM (Ply-NH^H150Y+I172T^) | This study |
| R6Δ*ply* | This study |
| R6:Ply^W433F^ | Surve *et al* (31) |
| R6:Ply-H:HlpA-GFP/tagRFP | This study |
| R6:Ply-NH:HlpA-GFP/tagRFP | This study |
