# Supplementary material for: Structural insights into loss of function of a pore forming toxin and its role in pneumococcal adaptation to an intracellular lifestyle
Source: PLoS Pathog. 2020 Nov 20;16(11):e1009016. doi: 10.1371/journal.ppat.1009016 (PMC7717573; doi:10.1371/journal.ppat.1009016)
Supplement: Attachment — Submitted filename: Response to Reviewers Critiques.pdf [file ppat.1009016.s019.pdf]

PPATHOGENS-D-19-01999

Point-by-point response to Reviewer critiques:

Part I: Major comments:

Reviewer #1:

1. In Figure 4C the authors suggest expression of ply NH in D39 leads to similar internalization when compared to ST306. The percentages shown here are 100-1000 fold lower than when the capsule is not expressed (0.005% recovery). The reviewer is slightly concerned with the number of bacterial cell recovery, which could be just left over bacteria in the wells not killed by the Abx treatment. Using microscopy in conjunction with the current data would be useful. Also, experiment in figure 4C possibly suggest a very mild effect of Ply-NH. Did the authors determine if the capsular production of D39 was being affected by the expression of NH ply?

Response: In our assays, antibiotic concentration and time has been optimized such that it is sufficient to kill 10x the SPN CFU used for invasion assays. But still, while performing gentamicin protection assays, we include a control well which contains only bacteria and no epithelial cells. This control is thereafter treated in the same way as the others culture wells in the assay and plated on agar after antibiotic treatment. We never recovered any viable bacteria following this protocol, suggesting that antimicrobial treatment is effective. Similarly, we have performed this assay with A549 cells infected with other serotypes and observed no invasion, suggesting that antibiotic is also effective when mammalian cells are present.

We agree that microscopic examination to show intracellular presence of SPN strain D39 would have been more convincing, but due to the sudden lockdown because of the COVID-19 pandemic, we are unable to perform this expt. But, our other experiments demonstrating co-localization of SPN with autophagy markers, clearly indicates intracellular presence of SPN. Moreover, in the gentamicin protection assays we ensured all extracellular bacteria were killed but still recovered CFU, confirming that bacteria were present inside the cell.

COVID-19 pandemic also interrupted the planned experiment to compare the capsule amount in D39:Ply-H and D39:Ply-NH, but we believe that the capsule content will be comparable between these strains as we did not perturb the capsule locus and the Ply locus is far away from the capsule locus in the SPN genome. Moreover, since our experiments in the background of un-encapsulated strain R6 demonstrated similar effects with respect to loss of pore forming activity of Ply, we trust that capsule will not play a major role in the observed phenotype.

2. In figure 5 the authors suggest that there is enhanced intracellular survival using A549 cells determined through testing R6 with various forms of ply. Lemon et al (mbio 2015 DOI https://doi.org/10.1128/mBio.02110-14) showed that when Spn is lysed through action of lysozyme present in the macrophage vacuole, it leads to ply release and its binding and oliogomerization causing bacterial products to be released into the cytosol, and eventual death of the macrophage. In the current scenario that would not happen with serotype 1 strain. Would it not kill the serotype 1 strain and not the macrophage? Did the authors test survival in macrophages? It would be useful if they did their experiments with either D39 or another clinical isolate, as the data would be much stronger with those strains.

Response: As per the suggestion by the reviewer, we have performed additional experiments to investigate interaction of SPN:Ply variants with macrophages. MTT assay was performed to determine the cytotoxic effect on R6:Ply variants on THP-1 macrophages (Fig S7C, page 48, lines 1112-1113). The MOI at which both R6:Ply-H and R6:Ply-NH strains of SPN were

found to be non-cytotoxic was further used to perform an intracellular survival assay in THP-1 macrophages, as shown in Fig 5B (page 44, lines 1013-1014). This corroborates our earlier findings in A549 cells and strengthens our thesis that loss of pore forming ability in Ply-NH promotes intracellular survival.
We, however, could not do the survival assay with D39 derivatives in THP-1 macrophages due to sudden lockdown imposed by COVID-19 pandemic.

3. The authors should test the D39 Ply- to provide a contrast between D39 with two different ply alleles. Does the NH allele behave more like a ply-? If so, it would also suggest that loss of ply could be beneficial under certain scenarios. Also did the authors observe any growth differences between strains that expressed different alleles of ply? As that could also compound interpretation of data.

Response: As suggested by the reviewer, we have now performed additional *in vitro* and *in vivo* experiments with a Ply-deficient SPN strain (D39Δ*ply* or R6Δ*ply*) and a non-hemolytic Ply variant (D39:Ply^{W433F} or R6:Ply^{W433F}).

In *in vitro* expts., although Ply knockout mutant (R6:Δ*ply*) failed to localize with Gal8 or ubiquitin (similar to R6:Ply-NH), a strain expressing a non-hemolytic Ply mutant, R6:Ply^{W433F}, co-localized with Gal8 and ubiquitin, albeit to a lesser extent than R6:Ply-H (Fig S9 A-C). This was also reflected in their intracellular survival capabilities wherein R6Δ*ply*, but not R6:Ply^{W433F}, demonstrated improved survival in A549 cells relative to R6:Ply-H (Fig S9 D-E) (page 48, lines 1129-1136). Interestingly, Ply^{W433F}, although non-hemolytic, has been speculated to form pores which causes sufficient damage to SPN containing vacuoles inside host cell, triggering recruitment of Gal8 and ubiquitin, resulting in autophagic killing, albeit to a degree lower than SPN expressing Ply-H. This is evident from our study as well as an earlier study, cited as Reference No. 29, and distinguishes Ply-NH, which has completely lost its pore forming ability, from Ply^{W433F}.

In *in vivo* expts., neither strains (D39Δ*ply* or D39:Ply^{W433F}) fully recapitulate the phenotype of D39:Ply-NH. Using a persistence mouse model (where we see SPN colonisation of lungs for at least 4 days in the majority of mice, with all strains used), we demonstrate that Ply-NH bearing strains D39:Ply-NH and ST306 colonised lungs at a higher density than those strains with Ply-H. By contrast, D39:Δ*ply* and D39:Ply^{W433F} colonised at a comparable density to the strain expressing Ply-H (Fig 6E). The SPN CFU count in lung tissue was compared to lavages, as a proxy for intracellular (or strongly adherent) and extracellular SPN, respectively. Higher numbers of bacteria were recovered in lavage than in post-lavage lung homogenates for all mice infected with D39:Ply^{W433F} or D39Δ*ply*, suggesting lung SPN are predominantly extracellular during infections performed with these strains (Fig 6G).

Hence, although *in vitro* SPN:Ply-NH behaves similar to a Ply knockout strain (SPNΔ*ply*) evading autophagy and demonstrating improved intracellular survival, the former exhibits an added advantage in colonization and persistence potential *in vivo* (in lungs). This leads us to the conclusion that expression of Ply-NH provides a benefit additional to that imparted by loss of its pore forming ability.

We also measured the growth kinetics of different strains (Fig S6D) and found that there is no significant difference between the strains tested that would explain the phenotypes observed.

Reviewer #2:

1. In vivo it is shown that the NH variant is less virulent, based on mortality data, leading to the conclusion that is promotes tolerance due to an intracellular lifestyle. If the NH version is better tolerated, why then do we still see improved clearance of this strain, which counteracts that statement? Could this be better assessed with a lower inoculum that would allow longer time frames to be examined for levels of bacterial burden to show the NH version facilitates persistence versus clearance by the H version of Ply? Alternatively, an in vivo model that better models persistence might be helpful.

Response: At the reviewer's suggestion, we have made efforts to develop an *in vivo* persistence model. This is challenging with strain D39 and its derivatives, but using a modified version of the method described by Haste *et al*. (Reference 35 in the revised manuscript), we were able to achieve colonisation of lungs over at least four days, with only around 20% of animals developing visible disease symptoms. The results of these experiments are shown in Fig 6D, E and G. The two Ply-NH harbouring strains (D39:Ply-NH and ST306) showed the highest density of lung infection at day 4 post infection and the highest CFU present in lung tissue homogenates, post lavage. We also incorporated additional Ply-derivatives into these experiments on the suggestion of Reviewer 1 and found that neither loss of Ply, nor loss of hemolytic activity (via W433F mutation) fully recapitulates the phenotype of the allele-5 Ply expressing strains.

2. There is also conflicting data between Figure 6C and 7B. In figure 6 after intranasal infection the authors show that ST306 is cleared more rapidly from the lungs at 24h, while there is a trend to reduced D39 Ply-NH vs H. Yet, in figure 7B (which doesn't give information on the infection in the figure legend) they try to make the point that there is a greater proportion of bacteria in the BALF. This figure is misleading as the supplemental data in S9 shows in fact that there is less bacteria in the BALF with ST306 vs the D39 strains. While the ratio of BALF to lung is different the absolute bacterial numbers are substantially less with ST306. This data does not support the notion that ST306 is more tolerated. How is this experiment in figure 7 different to 6? As bacterial burden BALF is usually reflective of that seen in lung tissue.

Response: We apologise that these experiments were not presented and described clearly. In the previous version of the manuscript, the data in the original Fig 6 are from whole lung homogenates (no lavage performed), whilst those in Fig 7 were from mice where lavage was performed prior to lung dissection and then bacterial burdens in the lavage fluid and the lung homogenate were separately determined. In our experience, bacterial numbers recovered from lavage are typically lower than those recovered from whole lung homogenates, although this may reflect differences in procedural technique. The reviewer is correct that overall CFU were lower in ST306 infection than in D39. We included Fig S9 (in the old manuscript) to be up front about this point. However, we agree with the reviewer that this is a limitation of the experiment. For this reason, we repeated this approach in the new persistence model we described above, performing lavage and lung dissection at 48 hours post-infection, a time when total lung CFU numbers were comparable across all infection groups (page 17, lines 355-357). These data are presented in Fig 6G. Only in the D39:Ply-NH and ST306 infection groups did we observe any mice where lung tissue CFU were higher than lung lavage CFU and in general the CFU recovered from tissue were higher in these groups than in D39:Ply-H infected mice. Whilst this does not directly demonstrate intracellularity (which is clearly shown in SBF-SEM and TEM images depicted in Fig 6F and Fig S11), we believe it supports our assertion of a more intimate host-pathogen interaction in these strains, with either strongly adherent or intracellular pneumococci.

3. Some of the in vivo conclusions are drawn from the comparison of D39 Ply-H to ST306 Ply-NH, which should be avoided since these are not isogenic strains and it has already been established through other assays in this study that they do have other factors that are different.

This is seen in the initial in vivo comparison as well as the microscopy, which should be done with isogenic strains.

Response: We agree with the reviewer, and have tried to limit these comparisons where possible. We wanted to be clear that D39:Ply-NH does not fully recapitulate the ST306 attenuated virulence phenotype, hence we believe it is valuable to include the initial comparison. Thereafter, we have tried to use D39:Ply-NH or not to compare the two Ply-NH bearing strains (D39:Ply-NH and ST306) wherever possible. We have included D39:Ply-NH in all *in vitro* and *in vivo* experiments except the TEM, where costs prohibited us from doing further experimentation. In this experiment, we felt it important to directly show evidence of *in vivo* intracellularity with ST306 and included D39 only as a means of demonstrating that this was not a phenomenon common to all SPN lung infection.

4. The authors throughout the study utilize a well-used strain of S. pneumoniae, D39, its WT Ply-H and the Ply-NH variant. Many of the assays performed would benefit by comparing these constructs to a straight Ply knockout strain as well as a toxoid variant (W433F). Does the ST306 Ply-NH version behave like a toxoid and/or just like a strain that doesn't express pneumolysin at all.

Response: We thank the reviewer for this excellent suggestion. As we have described in our answers above (to both this reviewer and reviewer 1), we have now included a Ply knock-out mutant and the Ply^{W433F} mutant in many of our *in vitro* and *in vivo* experiments. Neither strain fully recapitulates the phenotype of D39:Ply-NH in our persistence model (Fig 6E) although we know from the work of others that both these strains are attenuated in virulence in acute infection models. Moreover, our data suggest that though the W433F mutant is unable to cause hemolysis, it can form pores, which result in association of SPN containing vacuoles inside lung epithelial cells with "eat me" signals Gal8 and ubiquitin (Fig S9 A-C). This further drives SPN towards autophagic killing, as shown by higher intracellular survival of a Ply knock-out strain but not by a W433F mutant (Fig S9 D-E). We describe the significance of these findings in our answer to Reviewer 1, comment 3.

5. On page 8, Figure 1E, the authors introduce the concept that Ply-NH oligomers have an elevated appearance on the membrane, suggesting the pre-pore structures were not inserted into the membrane. This statement is not followed up by any experimental data.

Answer: Findings from our NBD experiment (Fig 3) suggest that in Ply-NH, the transmembrane β hairpins are not formed / inserted into the membrane. However, we agree that we do not have experimental proof to demonstrate the "elevated appearance" and hence have deleted that part of the sentence which refers to it (page 8, lines 143-144).

6. Mutating the residues in Ply-NH back to their respective Ply-H residues was able to restore activity when both mutations at 150 and 172 were reintroduced. Would a single substitution Y150H in Ply-H be sufficient to destabilize the pre-pore state and thus support the conclusion made at the end of the first paragraph on page 8 and Fig 2E.

Response: Among the mutations (Y150H and T172I), Y150H mutation is proposed to have a greater effect in loss of pore forming activity. Indeed, a replacement of Y150 with alanine (Y150A) has been demonstrated to result in reduction in the hemolytic activity of Ply to 0.2% of wild-type Ply (Kirkham *et al*; 2006). This reference is cited as Reference No. 36 in the text and has also been mentioned in the discussion (page 19, lines 387-388).

7. The non-hemolytic form of Ply appeared to improve invasion, initial uptake, of S. pneumoniae in different cell types. This difference was quite striking, yet by the 8h time point (Fig 5A) there is no difference, before then again showing a difference at 16h. How do the authors rationalize these results?

Response: Data in Fig 5A are expressed as a percentage of the viable CFU at 0 h (which is 2 h after addition of antibiotics to kill extracellular bacteria). So, absolute numbers of invaded bacteria are higher in R6:Ply-NH infection at both 0 (as the reviewer suggests) and 8 h, relative to other strains, but when data for all strains are expressed as a percentage of their 0 h numbers, this difference is not apparent. There is little killing within the first 8 h of infection. By 16 h, SPN CFU in both R6:Ply-H and R6:Ply-DM infection have declined more in both absolute (CFU numbers) and relative (%) terms, as compared to R6:Ply-NH infection. We decided to express the data as % of 0 h, to demonstrate that higher intracellular SPN with R6:Ply-NH is not simply a product of greater invasion efficiency, but also of increased relative survival intracellularly.

8. Figure 5 shows the presence/absence of autophagy proteins (galectin 8, LC3) and endosomal markers (LAMP-1) that are observed when cells are infected with the H but not NH versions of Ply. These images were taken at 10h post infection, which based on the invasion/persistence data is at a time point where there is no difference in bacterial numbers. Even when the bacterial numbers are different at 16 h it is a matter of ~15% vs ~9%. Are these observations meant to preclude the difference observed at 16h, even though they are imaged at 10h? Given this, further data is needed to make a conclusion that autophagy is at play here. This can be investigated using immunoblots to LC3B and/or using inhibitors to show that autophagy influences the persistence of the strains and that perturbing autophagy eliminates the phenotype between the two strains (e.g. rapamycin, cellular stress). Also Figure 5B, needs to include in the legend that DAPI is used to mark the nucleus otherwise people may think this is a large aggregation of bacteria, since bacteria is also labelled blue.

Response: At the onset, we would like to clarify that 10 h post infection (h.p.i) in the immunofluorescence experiments correspond to 8 h of the intracellular survival assay, since the 0 h for the latter begins after 2 h of antibiotic treatment.

In order to answer the concern, we have now included a time course analysis of ubiquitin co-localization to vacuoles containing R6:Ply-H and R6:Ply-NH (Fig 5E). Similar time-course of Gal8 co-localization was provided in the old manuscript (Fig 5D in revised manuscript). An additional analysis for Gal8 and ubiquitin co-localization, incorporating the control strains R6Δ*ply* and R6:Ply^{W433F}, at 18 h.p.i (corresponding to 16 h of intracellular survival assay) has been included in Fig S9 B-C. Another experiment demonstrating co-localization of SPN:Ply-H or SPN:Ply-NH containing vacuoles to Lysotracker (a marker for acidic organelles such as lysosomes) has also been included as Fig 5H. Overall, these suggest that the difference in association with autophagy markers evident at 10 h.p.i persist even at 18 h.p.i, resulting in differential association with Lysotracker. Following association with autophagic markers, further maturation, fusion with lysosomes and eventual degradation progresses with time, thus corroborating our results of early co-localization in immunofluorescence but differences in CFU at later time points in results of intracellular survival assay.

SPN:Ply-NH invades host cells in significantly higher numbers compared to SPN:Ply-H, and so we present these data with the initial counts at 0 h normalized to 100%. This is necessary to nullify the contribution from increased invasion towards intracellular survival (i.e. to determine the proportion of invaded pneumococci that survive, rather than absolute numbers of surviving pneumococci). Therefore, though the normalized difference in survival at 16 h is ~15% vs 9% (for SPN:Ply-NH and SPN:Ply-H, respectively), the difference in absolute CFU counts were significantly higher between these strains.

Though we have shown association of autophagy markers such as Gal8, Ubq and LC3 with SPN:Ply-H, we agree that inhibition of autophagy by 3-Methyl Adenine and subsequent intracellular survival assay could have emphasized its direct role in killing of SPN. However,

we could not perform this expt. due to the sudden and ongoing lockdown imposed because of the COVID-19 pandemic.

As suggested by the reviewer, the legend of Fig 5C (Fig 5B in the old manuscript) has been modified to mention that DAPI was used to stain eukaryotic as well as bacterial DNA (page 44, line 1016).

Reviewer #3:

1. Line 112-114 – "The resulting subdued inflammatory response permits establishment of a stalemate situation of host "tolerance" of SPN in the lower respiratory tract, favoring persistence for prolonged periods within an alternate niche." This represents a major conclusion of the manuscript. The authors have demonstrated that the non hemolytic form of ply promotes intracellular survival in cultured cells, but the in vivo data is less robust. Because the mice given D39WT strain die quickly, of course they can't demonstrate persistence compared with the ply-NH expressing strains. The data do not allow the authors to state that the persistence is "prolonged" since there is essentially no comparator. The data do support the hypothesis that loss of pore formation enhances bacterial uptake and survival within cells and, in mice, is associated with less inflammation and improved survival.

Response: We thank the reviewer for this comment, which is echoed by the other reviewers. We have removed reference to prolonged persistence in our description of the data from the *in vivo* models but have also demonstrated improved tolerance using an alternative infection model which lacks the acute inflammatory clearance that characterises our original model. These data are shown in Fig 6D, E and G and described in our responses to the reviewers above. These data show higher bacterial densities tolerated in the lungs of mice infected with D39:Ply-NH or ST306, compared to the other strains used.

2. Curiously, the authors do not use a ply deletion strain in any of their experimentation as a control. They show a clear difference between hemolytic and non hemolytic ply but cannot claim that any phenotype is due to actual expression of the non-hemolytic form without this control. See line 382-382 as an example "Our study demonstrates that the non-pore forming nature of Ply-NH provides an intracellular survival advantage to SPN, by enabling it to evade anti-bacterial autophagy." It is possible, given the lack of this control, that a ply deletion would show an identical phenotype to the D39 ply NH strain. This caveat should, at the very least, be discussed in the manuscript and any phenotype specifically attributed to expression of the non-hemolytic form removed. This is particularly true because a major emphasis of the paper is focused on the properties of the nonhemolytic form (that is binds to cholesterol and oligomerizes but does not insert). There are also issues with the D39/ D39NH comparison due to differences in antibiotic selection markers that do not appear to be adequately addressed (see below).

Response: As suggested by the reviewer, we have now performed additional *in vitro* and *in vivo* experiments with a Ply-deficient SPN strain (D39Δ*ply* or R6Δ*ply*) and a non-hemolytic Ply variant (D39:Ply^{W433F} or R6:Ply^{W433F}). The outcomes of these experiments and its significance has been described in response to Reviewer 1 comment 3.

Regarding the antibiotic selection markers, we have now transformed WT D39 with a construct containing the R6 *ply* ORF (which is identical to D39) and a spectinomycin resistance cassette. We then performed a comparison of the resulting transformant (D39:Ply-H) with the original D39 WT strain in an acute pneumonia survival experiment in mice (please see figure below)

and observed no differences in virulence between them. This strain (D39:Ply-H) was used in place of WT D39 for *in vivo* experiments.

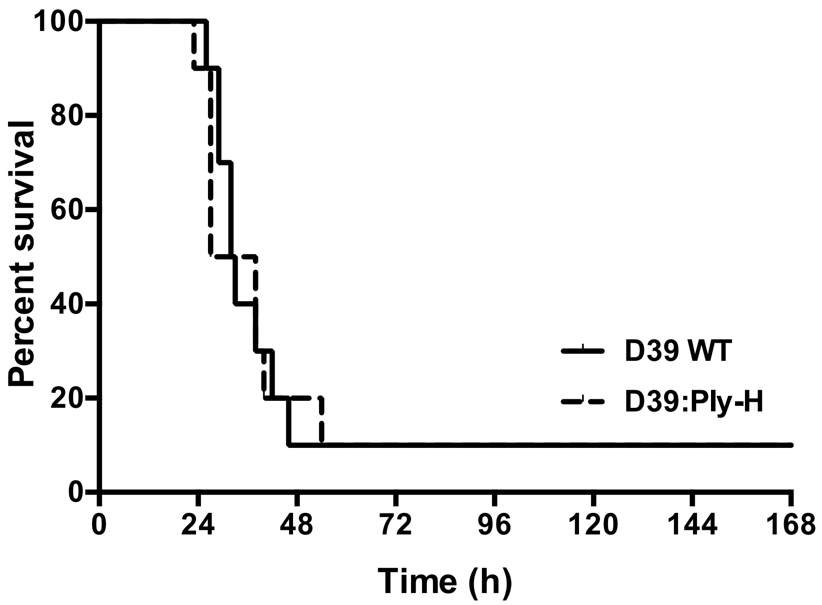

3. Mouse experiment – The comparison between D39 and D39-NH is the critical experiment here because the serotype 1 strain is likely to differ in many other ways from D39 that could influence the virulence phenotype. Given this, how did the authors assure that their generated D39 Ply-NH strain did not have unintended mutations or other changes related to transformation/ antibiotic cassette insertion? Did the authors compare opacity/transparency with the wt strain? Did they at least assess separate transformants? The strain was made with an antibiotic resistance cassette insertion; it does not appear that the D39:ply-H strain also carries an antibiotic cassette to address the possibility of this cassette resulting in the observed loss of virulence. The lack of an appropriately matched control should be addressed.

Response: As suggested by the reviewer, we have generated a D39:Ply-H strain by transformation of WT D39 with a construct containing the R6 *ply* ORF (which is identical to D39) and a spectinomycin resistance cassette. Whilst we used only a single transformant for the infection comparison shown above, we did perform growth rate analysis on 6 individual D39:Ply-H transformants and observed no differences as compared to D39. The growth data now included in the manuscript (Fig S6D) are from the transformant that we went on to use in the infection models. We observed no differences in opacity or transparency with the transformants tested. All our *in vivo* experiments are now performed with this newly generated D39:Ply-H strain to negate the effect of antibiotic cassette. Comparison of D39:Ply-H with D39 WT in a murine infection model confirmed that there is no effect of the antibiotic cassette on virulence (graph shown above).

4. Line 367 – "…increased propensity of Ply-NH containing SPN to embrace an intracellular lifestyle." The "propensity" here is based only on the ratio of lavage to tissue CFU and such may be overstated. It appears that if one compares the tissue levels between strains that they are not statistically different (no statistical tests are shown in the supplemental figure 9) such that the ratio is influenced by the overall cell density. In figure 7, similarly, the ratio is apparently only significantly different between D39 and ST306, not in the D39 and D39NH pairing. The most compelling data in this regard is in the comparison of EM identified intracellular

pneumococci after infection which the authors only see with the ST306 strain. Unfortunately, the authors did not assess the D39-NH strain in this way which, as above, seems to be the critical comparison.

Response: We performed the CFU counts in both the lavage and lung tissue in the new 4 day persistence model and observed the same trends. Mice with higher CFU counts in tissue than lavage were observed only in D39:Ply-NH and ST306 infections. We performed these analyses at day 2 post-infection, when total lung bacterial densities were comparable across all infection groups (page 17, lines 355-357). The EM experiments with ST306 was performed to show that the intracellular lifestyle we observed *in vitro* could be replicated in an infection model with a clinical strain. We agree with the reviewer that to have the comparable images for D39:Ply-NH would be ideal, but the cost of these EM experiments was prohibitive. We have searched exhaustively for comparable examples of intracellularity in D39-infected lungs and have found none. We hope that the *in vivo* demonstration of intracellular ST306, together with our in vitro data using D39:Ply-NH, are sufficient to demonstrate that allele 5 Ply is key to promoting intracellular survival.

5. Figure 5B – How do the authors know that the ply-NH strains in this assay are intracellular? I'm not actually sure how they are identifying the bacteria since they seem to lack any label. Line 459-462 – suggests that the authors are using fluorescent strains but there is no mention of these anywhere else in the manuscript. The images and methods are not clear on this at all, the strains are not labeled as GFP or mCherry (just SPN) and there is no information about strain background in the legend or methods. If they are used, they are confusingly colored blue, identical to the nuclear staining of eukaryotic cells. If the bacteria are labeled, can the authors explain how they confirm that the cells are intracellular since no label for a non autophagic vesicles is used? The NH bacteria are almost impossible to see in the provided figure.

Response: For quantitative immunofluorescence experiments (Fig 5D-H) either GFP or tag-RFP tagged SPN were used for infection, or unlabelled SPN was stained with anti-Enolase primary antibody post fixation. For these experiments, intracellular SPN was distinguished from extracellular SPN by differential staining. Extracellular SPN was stained prior to permeabilization; another round of staining post-permabilization yielded the extracellular SPN dually stained while intracellular SPN remained single stained. This has now been described in Materials and Methods under the heading Immunofluorescence (page 32, lines 678-681).

Fig 5C in the revised manuscript (Fig 5B in the old manuscript) is a representative image showing co-localization of different autophagy markers to vacuoles containing SPN:Ply variants. Since all the different markers are shown in a single image, we had to use DAPI to stain SPN (which also stains the host nucleus). Intracellular SPN in this case was identified by confirming that the z-stack containing the SPN of interest is indeed part of the intracellular compartment (visualized as diffuse cytosolic staining of the respective markers). We have now included the z-stack numbers of the representative confocal images in the legend of Fig 5C which clearly demonstrate that SPN was intracellular (page 44, lines 1017-1019).

Part II – Minor Issues: Editorial and Data Presentation Modifications

Reviewer #1:

1. Ln 52 the authors mention that ply is secreted. To date no signal sequence for ply has been determined. Current understanding suggests that when Spn autolyses, and ply is released into the host environment. The authors should correct it. The authors might also want to mention in summary or introduction that the inflammatory response in the nasopharynx, also leads to eventual clearance through macrophages.

Response: We thank the reviewer for noticing this error. We have changed the word “secretes” to “expresses” in the Author summary and added a brief discussion on inflammatory clearance of SPN in the nasopharynx (page 5, lines 85-88).

2. Within the introduction the author should bring up the point that loss of ply leads to increased persistence within an animal model and recently shown to be mediated through IL1 signaling (Kuipers et al 2018 PLOS Pathogens, https://doi.org/10.1371/journal.ppat.1007396) which is the one of the general premise for the study in regards to serotype 1 strain and its ply non-hemolytic nature.

Response: As suggested by the reviewer, we have mentioned the role of Ply in IL1 signalling and clearance of SPN in the nasopharynx, in the introduction (page 5, lines 85-88; Reference No. 10). We have also made reference to the earlier work of the same group: Matthias *et al*, J Immunol, 2008 (page 5, lines 85-88, Reference No. 11), which demonstrated that Ply increases clearance of colonisation in a mouse model.

3. The authors should carry out fisher’s exact test (2 x 2 contingency test) between survivors between D39 NH and ST306 (Figure 6C). From the data it appears that there are more survivors in serotype 1 isolate. The survival data would suggests that even with D39 expressing NH ply, it is present in higher numbers (Figure 6 C @ 48 hours) and survives poorly compared to ST306. These data would clearly suggest that there are other factors besides ply that contribute towards the phenotype of ST306, and the authors should try to incorporate that into their current study.

Response: We agree with the reviewer on this point and acknowledged the contribution of other factors to attenuated virulence of ST306 both in the original manuscript and in this revised draft (page 15, lines 313-317). On the reviewers suggestion, we have added the p-value for the ST306 vs D39:Ply-NH Fisher’s exact test to the text ($p=0.0442$).

4. In Ln 408-411 authors mention that serotype 1 isolates might transmit via disease state. Are they suggesting a different route of transmission for type 1 compared to others? If so isn’t the central premise of the authors that type 1 isolates have attenuated virulence, thus would cause less disease?

Response: What we are hypothesising here is that ST306 might cause milder respiratory disease, something consistent with the epidemiological pattern, whereby Europe has seen a number of outbreaks caused by ST306, with the majority of cases being non-lethal (Jefferies JM, JID, 2007; Reference No. 13). We believe that though virulence is attenuated, some residual disease-causing capacity is required to stimulate transmission, by, for example, coughs and sneezes. In our acute murine infection model, we did see occasional disease (~10% of mice) upon infection with ST306, so although attenuated, we do not consider it an avirulent strain.

5. The reference 37 in line 417 appears to be in incorrect. That reference does not provide

any data on colonization potential of serotype 1 strains. This reviewer believes the article by Birgitta Henriques-Normark group “Virulence in Mice of Pneumococcal Clonal Types with Known Invasive Disease Potential in Humans” should be cited, which mentions colonization density of type 1 isolates and suggests that they colonize poorly. https://doi.org/10.1086/432513. If the authors have another pertinent reference it should be cited here.

Response: Reference 37 in the old manuscript (Reference No. 48 in the revised manuscript) talks about the poor transformability of serotype 1 isolates, which we mentioned in the same sentence in the old manuscript. We have, however, moved the placement of this reference to make it clear which part of the sentence we were referring to. We have also included the reference suggested by the reviewer in the revised manuscript (Reference No. 33 in page 21, line 450).

Reviewer #2:

1. There are no statistics for figures 1, 2, 3 and 5C.

Response: As suggested by the reviewer, statistical significance have been incorporated in above mentioned figures (Fig 1A, 2D, 2G, 5).

2. In figure 7B is D39 Ply-NH different to H?

Response: These data have been replaced by the comparable experiment in the four day persistence model. Adopting this new infection model, at 2 days post-infection (chosen since total lung CFU in all groups were comparable at this time point), 2/5 D39:Ply-NH infected mice had a higher number of bacteria in post-lavage tissue homogenates than in the lavage itself (Fig 6G in the revised manuscript). By contrast, all mice infected D39:Ply-H had higher numbers of bacteria in lavage than in post-lavage lung homogenates.

3. Figure 4D and E, what cells are used?

Response: In both these experiments A549 cells were used. This has now been mentioned in the legends of Figure 4 (page 43, lines 997-1001).

4. Figure 5 is lacking an uninfected control.

Response: There will be only diffuse signal of Gal8 or other autophagy markers in the cytoplasm of uninfected cells, similar to that observed upon infection with SPN:Ply-NH. Therefore, inclusion of an uninfected control in the co-localization experiments shown in Figure 5 would not lead to any major conclusions.

5. Figure 7, is this a single experiment or was it repeated?

Response: The EM was performed on lungs from two mice infected with D39 and two infected with ST306. The inoculum was prepared independently for each mouse, on sequential days (i.e. 1x D39 and 1x ST306 on day 1, 1x D39 and 1x ST306 on day 2). Number of animals tested in this experiment were limited due to costs, but we have searched exhaustively for intracellular in SPN in EM images from D39-infected lungs and found none. By contrast, we found multiple examples of SPN within cells in both ST306-infected mice.

Reviewer #3:

1. Figure S2 really doesn't add anything, a list of alleles with their mutations from wt noted or an alignment would be more informative. Additionally, some designation regarding what % of serotype 1 strains have been found to have each allele would be helpful given the author's hypothesis that this is a major phenotype of this serotype.

Response: We considered the point raised by the reviewer. However, showing alignment of 20 alleles of Ply spanning 471 amino acids will make it an extremely large figure. Alternately, a table listing the amino acid differences between all the 20 alleles of Ply is already available in Yun *et al*; 2015 (Reference No. 15). We therefore are of the belief that retaining phylogenetic tree is best way to showcase the difference between Ply alleles. However, we have now mentioned the amino acid mutations with respect to wild type allele 1, against each of the other alleles, in parenthesis.
Information regarding serotype 1 strains harbouring different Ply alleles is already available in Jefferies *et a*l; 2007 which is cited in Reference No. 13.

2. Figure 1C really doesn't add anything since ply-NH doesn't lyse RBC. This figure could be removed or moved to supplemental since the binding to ghost cells experiment addresses the question of whether the NH version interacts with cholesterol.

Response: As per suggestion of the reviewer, we have removed the old Fig 1C. Since this did not add anything new to manuscript and could not be coupled to any other supplemental figures due to thematic differences, we have removed this figure from the manuscript.

3. Line 143 "Interestingly, Ply-NH also formed rings of similar size to Ply-H, which was further confirmed by SDS-PAGE, following treatment of eukaryotic membranes with the Ply variants." The pores do not look the same size in this figure, NH generated pores look quite a bit bigger. I don't believe that the western blot could help to resolve a difference in the number of ply molecules given the large size of the band. I'm also not sure what the authors mean about an "elevated appearance". If they are referring to the increased contrast with some of the rings then it would mean that many rings without this contrast are actually inserted into the membrane which is contrary to their hypothesis and subsequent data. This point needs clarification. Can the larger size be explained by the crystal structure? Could it be that a larger oligomer is actually the reason for the loss of hemolysis (some sort of instability)?

Response: Both the samples of Ply-H and Ply-NH were prepared in similar manner for electron microscopy (EM) analysis and pictures were taken at constant or given magnification (indicated by scale bar). We agree with the concern raised by reviewer that the elevated appearance of the Ply-NH pore is not convincing from the two dimensional image. Also the elevated appearance cannot be explained by crystal structure as Ply-NH molecules are present in crystal lattice, in contrast the EM pictures shows the arrangement and oligomerization of Ply-NH monomers on the model membrane with cholesterol; hence we have removed the statement from the revised manuscript (page 8, lines 143-144).

4. Line 222 – Table S2 is incorrectly referred to here. I believe it should be table S3.

Response: Table S2 is correctly mentioned in this refereed place as it depicts the relative hemolytic activities of cysteine substituted mutants of Ply-H and Ply-NH, before and after labelling with NBD dye (page 11, lines 218-219).

5. Line 223-4 – It's not clear why the authors chose to change different amino acids in the ply-H and ply-NH derivatives. Using different amino acids does not allow them to determine if the cysteine changes directly affected the structure of the NH strain since it is non hemolytic to

begin with. Can the authors explain why they did this and how they can ensure that the cysteine changes didn't affect the native structure of ply-NH?

Response: In order to address the concern of the reviewer on "selecting different amino acids for Ply-H and Ply-NH"; we have created three new Ply-H (H184C, D257C and E260C) mutants which are identical to Ply-NH cysteine mutants. We have presented fluorescence experimental data of those NBD dye labelled mutants in modified Fig 3. To rule out the possibility of change in the native structural folds of the cysteine substitution mutants of either Ply-H or Ply-NH, we performed hemolysis assay of the mutants and labelled proteins (Table S2). Interestingly, they do not have significant effect on their hemolytic properties upon cysteine mutation and labelling. So, we are sure that the cysteine mutants of Ply-H and Ply-NH have native structural fold.

6. Line 239 – Fig S6C is referenced but there is no information in this figure that pertains to the preceding sentence.

Response: Fig S6C depicts that Ply expression level is similar between different SPN strains (D39:Ply-H, D39:Ply-NH and ST306). As invasion experiments were performed with different SPN strains, and Ply plays a role in cellular invasion, it was important to demonstrate that all strains express Ply at similar level and that the differences observed in invasion were due to loss of pore forming activity of Ply and not due to difference in its expression. The reviewer is right to point out that this was not correctly referred to in the text. This has now been corrected in the revised manuscript where the D39 Ply allele exchange variant is first introduced (page 12, lines 235-237).

7. Line 306-307 – I'm assuming these are total CFU, reflecting what would be obtained with lavage + tissue? It would help to state this since the authors go on to assess each compartment separately.

Response: Fig 6B depicts CFU obtained from whole lung homogenates of mice without any pre lavage extraction procedure. This is now mentioned in the text (page 15, lines 306-309).

8. In S9AG, the x axis label is incorrect (hours).

Response: This figure has been completely re-worked, with the addition of new strains. It is now part of a main figure (Fig 6G) and the error has been corrected.

9. Line 344 – the word "substantial" is highly subjective given no comparator.

Response: We have replaced this with "residual" (page 18, line 372).

10. Line 408 – "Indeed, the association with outbreaks may imply that the primary transmission route is disease-dependent, rather than occurring during asymptomatic nasopharyngeal carriage." This still doesn't explain the persistence of NH serotype 1 strains in the population between outbreaks. Wouldn't a more compelling hypothesis be the serotype 1 strains occupy an intracellular niche (maybe within the nasopharynx) and are "missed" when individuals are surface swabbed for colonization status?

Response: We thank the reviewer for this excellent suggestion. We have added a line to discuss this hypothesis (page 21, line 441-444).
