# Supplementary material for: Structural insights into loss of function of a pore forming toxin and its role in pneumococcal adaptation to an intracellular lifestyle
Source: PLoS Pathog. 2020 Nov 20;16(11):e1009016. doi: 10.1371/journal.ppat.1009016 (PMC7717573; doi:10.1371/journal.ppat.1009016)
Supplement: Attachment — Submitted filename: Response to Reviewer Comments.pdf [file ppat.1009016.s020.pdf]

PPATHOGENS-D-20-01305

Response to Reviewer Critiques

Part I - Summary

Reviewer # 1

1. The authors provide a crystal structure of the pore-forming toxin, pneumolysin, from strain ST306, a strain Streptococcus pneumoniae that produces a non-hemolytic (NH) variant of pneumolysin. They go on to present data to suggest that this NH variant leads to increased phagocytosis of S. pneumoniae and facilitates intracellular persistence. In vivo, the NH variant is more rapidly cleared but also invokes a reduced inflammatory response. The study presents the new crystal structure for this non-hemolytic variant of pneumolysin, which is of interest to the field. Where this study falls short is the inconsistencies with the in vitro and in vivo data. Many of the observations seen are largely what is observed with pneumolysin knockouts or toxoid variants, and thus do not add significantly to the field. New data shows that the NH version can persist in a low dose model, the mechanism for which is not known, which would be the major shortfall of the study, when other data is limited as far as comparisons to toxoid or clean deletion strains. The major mechanistic advance would be to understand why this pneumolysin expressing versions persists better than the other variants.

Response: We thank the reviewer for positive comments and constructive criticism of our work. We are appreciative of this process which has helped to improve the manuscript. In the revised manuscript we have attempted to address the concerns raised by the reviewer and have added new data with ply knockout strains and the W433F toxoid variant. These new data demonstrate that possession of Ply-NH is not comparable to either loss of Ply (see comparisons with the knockout strain) or presence of a non-hemolytic Ply (see comparisons with W433F strain), in terms of infection outcomes.

It has been demonstrated previously (Reference # 7) that, at low concentration, Ply interacts with MRC-1 on dendritic cells (DCs) to impede secretion of pro-inflammatory cytokines. We now show that DCs secrete higher amounts of pro-inflammatory cytokine TNF-α when infected with a Ply deletion mutant compared to an isogenic Ply-NH-bearing SPN strain (Fig S12A). This finding offers some insight into why D39:Ply-NH derives an advantage relative to D39Δ*ply in vivo*, as it would be subjected to reduced downstream inflammatory effects, such as macrophage killing.

SPN strains harbouring different Ply variants are differentially susceptible to intracellular killing mechanisms upon invasion of lung epithelial cells. A Ply^{W433F}-bearing strain associated more readily with autophagy markers than did D39:Ply-NH (Fig S9B-C and E), and this finding may explain the relative *in vivo* advantage of D39:Ply-NH vs D39:Ply^{W433F}.

Reviewer # 2

1. The article submitted by Badgudgar et al represent a highly ambitious study on the impact of non-hemolytic pneumolysin on serotype 1 disease. The authors go on to crystalize the non-

hemolytic version of pneumolysin found in serotype 1 ST306, determine that it binds to the cell, forms oligomeric rings but does not insert into the pore due to a mutation in the area that forms transmembrane b-hairpins (Y150H and T172I, that this mutation (modestly) prolongs the ability of pneumococci to survive within cells, and despite being attenuated overall for disease pneumococci that express a non-hemolytic toxin are able to persist within what appears to be an intracellular state. The conclusions reached by the authors are that this represents an evolutionary move by the bacterium to co-exist with the host as otherwise it causes death which is not optimal for the bacterium. The authors are to be commended in what is truly a herculean effort in regards to the structural aspects of the work. Unfortunately the rigor drops off as they move towards the evidence that suggests this intracellular state is vital for the disease process and that this is a major aspect in the lifestyle of serotype 1, moreover that the ability to persist in vivo is driving type 1 disease.

Response: We thank the reviewer for appreciating our efforts and have performed additional experiments, both *in vitro* and *in vivo*, to strengthen our claims. We believe with these additional experiments can address the concerns raised by the reviewer. Comparison of interaction of Ply-NH harbouring SPN with THP1 macrophages versus A549 alveolar epithelial cells indicate that though the invasion is higher in the macrophages (presumably due to phagocytosis), the survival and recycling efficiencies are significantly less compared to A549, particularly at later time points (Fig S13). Thus, a niche inside non-professional phagocytes, such as alveolar epithelial cells, would ensure the improved survival of SPN and additionally enable its egression out of the cells for the purpose of transmission.

2. One other major concern is that the authors repeatedly simplify the complexity that is pneumococcal disease. A much more nuanced statement of how pneumococcal disease arise, the role of colonization and inflammation, and disease is required.

Response: As suggested by the reviewer, we have expanded the introduction to acknowledge the role of Ply in transmission, in dampening immune responses via MRC-1 interactions, and in stimulating inflammation and disease via lysis of host cells (Page no. 5, lines 86-88 and 92-97).

3. Overall the data that suggests intracellular persistence of type 1 ST306 is vital for prolonging the bacterium state and this confers an advantage to the bacterium is insufficient. I say this not to detract from what in many ways is an excellent work effort, but to make sure that the conclusion reached by the authors are well supported and therefore find the same support by future readers. As is, I remain skeptical and I would dismiss the papers conclusions as being unsubstantiated.

Response: We thank the reviewer for appreciating our efforts and trust that the revised version provides sufficient evidence to convince that the intracellular residence of serotype 1, aided by Ply-NH, plays a significant role in its persistence. Specifically, we have added the following data:

1. New acute infection model data to demonstrate that infection progresses differently in D39:Ply-NH infected mice than in those infected with wither D39Δ*ply* or D39:Ply^{W433F}, suggesting that harbouring allele 5 pneumolysin is not functionally equivalent to either loss of pneumolysin or possession of a non-hemolytic toxoid variant (Fig 6A, B).
2. A comparison of intracellular survival and egression of Ply variants in airway epithelial cells and in macrophages, demonstrating prolonged survival and egression of Ply-NH in airway epithelium (Fig S13).

3. TNF-α production by DCs infected with D39:Ply-NH or D39Δ*ply*, demonstrating higher production in the latter. This suggests D39:Ply-NH may possess some residual MRC-1 binding that may aid persistence via dampening of immune responses *in vivo* (Fig S12A).
4. Evidence of intracellular pneumococci in D39:Ply-NH infected mice (SBF-SEM images), allowing a direct comparison with D39:Ply-H (Fig S12B and S1 Movie).

Reviewer # 3

1. The current study by Badgujar et. al concerns the pore forming toxin pneumolysin (Ply) and the structural difference between traditional Spn strain and serotype 1 isolates that are non-hemolytic. This is a resubmission of the original submission. The first part of the study is based on the solved crystal structure of the NH Ply by the authors, and provides interesting contrasts with the hemolytic-Ply variants. One of the distinguishing features that makes Ply-NH unique is its ability to bind and oligomerize on cell membranes but not to form pores. Next, the authors test the differences between the two Ply in different cell culture models and establish that the loss of pore formation leads to increased internalization and persistence of Spn in host cells. Lastly, the authors test the difference in ply in a murine model, and suggest that defective NH ply strains are less virulent and persist longer in the host.

Response: We thank the reviewer for positive comments regarding our work and for providing a summary of our findings.

Part II – Major Issues

Reviewer # 1

1. Phagocytosis was performed with R6-Ply-NH and -H. How does this compare with a Ply null or toxoid derivative? The authors also mentioned differences were not due to differential pneumolysin production. Isogenically derived strains of S. pneumoniae do exist with altered Ply production, if this were to be tested.

Response: As suggested by the reviewer, we have performed phagocytosis assay with the Ply null mutant. We observed that at extended time points post infection, both ply mutant and Ply-NH bearing strains, showed improved persistence compared to SPN strain bearing Ply-H (Fig S9F).

We have shown by western blotting and ELISA that isogenic SPN strains harbouring various forms of Ply express Ply in comparable amounts (Fig S6A-B). As our study focusses on how structurally different form of Ply influence interactions with the host, we believe a comparison of different levels of Ply production would be beyond the scope of this manuscript.

2. In vivo the authors show there is improved survival of mice with the NH variant, lack of Ply is known to influence virulence. The capacity to persist was seen when bacterial counts were determined at later times (Figure 6B)-no statistics is shown in this, is NH vs H statistically different? Is this result any different to a Ply null strain? These mice no doubt have a reduced host response.

Response: As only 1 mouse infected with Ply-H bearing SPN strain survived till later time points, we are not able do a statistical comparison with Ply-H. Instead, we have now added data for both D39Δ*ply* and D39:Ply^{W433F} and show statistical comparisons vs D39:Ply-NH (Fig 6B). In this model, Ply-H infected animals succumb early, likely due to overwhelming inflammation. However, animals infected with Ply-NH, Δ*ply* or Ply^{W433F}-expressing strains survive longer, with only 0-20% of mice developing invasive disease. At one-week post infection, surviving mice infected with D39:Ply-NH had significantly higher lung CFU than those infected with either D39Δ*ply* or D39:Ply^{W433F}. We believe that the explanation for this finding is as follows:

1. D39Δ*ply* cannot interact with MRC-1 and so stimulates cytokine secretion from DCs, whilst D39:Ply-NH retains residual MRC-1 binding activity (Fig S12A). Ply's interaction with MRC-1 have been shown to impede fusion with lysosomes and thwart pneumococcal killing (Reference # 7).
2. D39:Ply^{W433F} is less able to avoid autophagy pathways in airway epithelial cells than D39:Ply-NH (Fig S9).

3. Figure 6E would appear to be an important piece of data that delineates Ply-NH vs the toxoid mutant and a Ply null strain. The question is why does this behave differently?

Response: We hope our answer above goes some way to addressing this question. We believe that Ply-NH has an autophagy-evasion advantage relative to D39:Ply^{W433F}, enabling persistence intracellularly, and an immune response dampening advantage relative to D39Δ*ply*, by virtue of interactions with MRC-1.

Reviewer # 2

1. Introduction, The authors paint a black and white picture of pneumococcal disease the bacterium is a killer. This is a simplification that is not true, the vast majority of individuals who are colonized are asymptomatic. Severe disease is in large part due to the state of the host, which is why Spn is generally considered to be an opportunistic infection. Most serotypes of Spn, the exception being type 1, have very low attack rates and do not with any frequently cause disease. Thus despite producing a hemolytic version of pneumolysin the some versions of this bacterium have adapted for long-term asymptomatic colonization. The idea that the bacteria has an attenuated toxin to reduce disease severity and prolong duration is therefore perhaps one that is restricted to serotype 1 versus being generally applicable to all of Spn.

Response: We apologise for this unintended oversimplification. We had altered the emphasis within the introduction, making changes at lines 71-73 and adding new text (lines 92-97) that highlights the key role played by hemolytic Ply in ensuring onward transmission. This concept is revisited in the discussion (Page no. 24, lines 493-496) and we have added text here to indicate that we are referring specifically to serotype 1, with regards to the advantages of Ply-NH.

2. Lines 80-87, Pneumolysin triggers lung epithelial cell death via necroptosis, this has been demonstrated to cause the release IL-1α from necrotic cells which is inflammatory. This should be added to the text.

Response: As suggested by the reviewer, Ply mediated necroptosis of lung epithelial cells and subsequent release of IL-1α has now been described in the text (Page no. 5, lines 86-88).

3. I found the structural data to be strong and have no concerns. The authors should be commended on a very detailed and robust bit of work. The supplemental data provided by the investigator is also excellent and I congratulate the authors on their efforts to provide clarity and transparency in this manner.

Response: We are glad to know that the reviewer liked our work and thank him/her for appreciating our efforts.

4. Line 243, The authors hypothesize that it is the targeting of PLY-H on lipid rafts that is the reason why PLY-H impairs endocytotic processes whereas PLY-NH does not. Indeed PLY is likely to be found on lipid rafts as these are cholesterol rich and support the oligomerization of the toxin-ultimately causing them to be dispersed. However, it is seems far more likely that the ion-dysregulation caused by the toxin-formed pores formed by PLY-H is responsible for altering cell signaling within the cell, both due to the ion dysregulation but also energy loss in attempt to resotore the ion gradient. There is also a patch/plug response that will disrupt other normal signaling and endocytosis events – as pore-complexes are expelled. Thus the impact of hemolytic pneumolysin on a cell is very broad with lipid raft formation being only one aspect. This is not adequately recognized.

Response: We agree with the reviewer that the impact of the pore forming ability of Ply-H on host cell is multifaceted. Apart from its effect on the lipid raft, pores formed by Ply-H on the plasma membrane can cause ion imbalance and disrupt other normal cellular signalling mechanisms which in turn could hinder endocytosis events. This has been now been acknowledged in the text (Page no. 21, lines 419-422). However, disruption of lipid rafts by Ply mediated pores appears to be a key reason for the reduced invasive ability of Ply-H harbouring SPN which we investigated in the present study.

5. The results on prolonged intracellular survival in A549 cells shown in Fig5A (a difference at 16h of 9.5% to 13.5%; at 24h 2.5% to 6%) (are less than compelling). ON the other hand the data shown in Fig 5B with macrophages are more so. With data from the Oggioni lab now demonstrating that Spn replicate intracellularly and emerge, what is the fate of the bacterium and the cells that are hosting them? I think its important for the authors to consider that intracellular persistence within epithelial cells may not be why serotype1 does this and the effect may be principal in other cell types and affecting measures not examined. The fate of the hosting cells may provide an important clue.

Response: As suggested by the reviewer, we have now performed invasion, intracellular survival and recycling assay on both A549 and THP-1 cells following infection with SPN strain bearing Ply-NH (Fig S13 A, C-D). Our results suggest that though the invasion is higher in THP-1s (due, presumably, to its higher phagocytic uptake ability), the survival and recycling efficiencies are significantly less compared to A549, particularly at later time points. This demonstrates that though there may not be a preference in terms of invasion in a particular cell type, SPN invading the epithelial cells have higher chances of survival and egression as compared to those invading macrophages. We also tested the fate of both the host cell types following pneumococcal infection and found that there is no significant difference in the host cell viability before and after infection with a Ply-NH bearing SPN strain (Fig S13B).

6. Figure 6F. Given the importance of the finding to the authors claim, the authors need to provide quantitative data on the number of intracellular pneumococci they detect within infected lungs. As

the authors are adept at immunofluorescent staining this requires verification with a co-stain that the bacterium are indeed intracellular.

Response: We appreciate the reviewer's suggestion regarding this point, but we have not provided quantitative data from the electron microscopy experiments due to the nature of this analysis. Due to the cost of the SBF-SEM and TEM, we were restricted to analysis of lungs from D39:Ply-H, D39:Ply-NH or ST306-infected mice, each taken from a single animal. We analysed lung slices taken at random throughout the lung and, where we observed intracellular pneumococci, we then analysed successive 180 nm sections in order to build up a 3D image of the bacteria within cells, confirming their size, shape and intracellularity (added as S1 Movie). We observed pneumococci repeatedly in ST306 infected lungs (7 separate SPN clusters from analysis of four 1 mm^3 lung sections) and D39:Ply-NH infected lungs (12 separate SPN clusters from four 1 mm^3 lung sections) but found no comparable structures in D39:Ply-H infected lungs (ten 1 mm^3 lung sections analysed). We felt that we could not accurately extrapolate from these numbers to estimate total intracellular numbers in the whole lung, as the bacteria would not likely be evenly distributed throughout the lung and to section the entire lung and perform in this way would be prohibitively expensive and time consuming to analyse. However, we analysed additional D39:Ply-H sections, from other areas of the lung, to rule out the possibility that we had missed the part of the lung in which pneumococci resided. We hope the reviewer will understand the technical and financial challenges associated with these experiments and that the additional movie helps convince that the SPN are intracellular. The 3D movie constructed from SBF-SEM images of a D39:Ply-NH infected mouse lung is shown in S1 Movie and one of the sections depicting presence of SPN is shown in Fig S12B.

7. Demonstration of more recoverable Spn in surviving mice infected with D39-NH versus those that are dead is not really the same thing as showing this is an advantage for the bacterium in evolutionary manner.

Response: We agree with the reviewer on this point. In the acute model, we compared CFU only between surviving animals, but accept that this analysis is skewed by different numbers of mice lost to lethal infection in each group. We have included the persistence model data to deal with this issue.
Likewise, we agree that demonstration of elevated lung CFU is not sufficient to conclude there is an evolutionary advantage. We proposed the evolutionary argument merely as a hypothesis for consideration by the field and as a suggestion for future endeavours. However, as per suggestion of the reviewer. we have modified the language in the text to make this distinction clearer (Page no. 24, lines 493-496).

Reviewer # 3

1. One the major concerns that the reviewer had was in regards to Figure 4C. Where the authors recovery rate is very few cells. The reviewer had suggested that authors carry out microscopy, which the reviewers were unable to do because of Covid-19. With such low recovery it would be beneficial to show some further data with this assay, as the ST306 is no different than WT D39. To alleviate concerns did the authors carry out this assay using an R6 with the two different variants in this assay? It would be predicted that NH would have increase intracellular numbers compared to H variant.

Response: As suggested by the reviewer, we have performed the invasion assay on primary human pulmonary alveolar epithelial cells with R6:Ply-H and R6:Ply-NH strains. R6:Ply-NH strain exhibited significantly higher invasive ability compared to R6:Ply-H strain (Fig 4C).

2. In figure 6F the authors show intracellular Spn with ST306, but none were observed with D39 H. They predict that part of the reason for that is due to NH ply variant. An important control that is missing here is the D39 NH variant, where they should be able to see intracellular bacteria.

Response: As suggested by the reviewer, we have performed serial block face imaging on mice lungs infected with D39:Ply-NH. Our results suggest that unlike D39:Ply-H, D39:Ply-NH is found inside lung epithelial cells, similar to ST306 (Fig S12B and S1 Movie).

Part III – Minor Issues:

Reviewer # 1

No comments

Reviewer # 2

1. Line 52. Pneumococcal genes are expressed, proteins are produced. Spn triggers inflammation by elaborating pneumolysin not expressing it.

Response: As suggested by the reviewer we have now corrected the referred sentence (Page no. 3, line 52). We have also corrected the same in Page no. 4, line 80 (expressed changed to produced) and Page no. 5, line 98.

Reviewer # 3

1. Figure 6D and G are missing any statistical analysis.

Response: This has been corrected (Page no. 48, lines 1105-1106 and 116-1117).
